# Correlations between problematic internet use and suicidal behavior among Chinese adolescents: a systematic review and meta-analysis

**DOI:** 10.3389/fpsyt.2024.1484809

**Published:** 2024-11-11

**Authors:** Xubin He, Si Chen, Qinyao Yu, Ping Yang, Bo Yang

**Affiliations:** ^1^ Chongqing Mental Health Center, Chongqing, China; ^2^ Chongqing Medical School, Chongqing, China

**Keywords:** problematic internet use, suicidal Behavior, adolescents, China, meta-analysis

## Abstract

**Background:**

Problematic Internet Use (PIU) has been increasingly linked to suicidal behavior among adolescents, raising significant public health concerns, particularly in Chinese youth. This study aimed to systematically review and meta-analyze the correlation between PIU and suicidal behavior in Chinese adolescents to provide a clearer understanding of this association.

**Methods:**

A comprehensive search was conducted across seven databases up to July 1, 2024. Studies investigating the relationship between PIU and suicidal behavior among Chinese adolescents were included. A random-effects meta-analysis was employed to assess pooled effect sizes, with subgroup analyses conducted to explore potential moderators, such as geographic region, age, gender, assessment tools for PIU and suicidal ideation, and the presence of depression. Data analysis was performed using STATA software (version 16).

**Results:**

This meta-analysis, comprising 23 studies with 353,904 participants, identified significant associations between PIU and suicidal behavior among Chinese adolescents. PIU was associated with increased risks of suicidal ideation (OR = 1.72, 95% CI: 1.45, 2.03), suicidal plans (OR = 1.50, 95% CI: 1.02, 2.20), and suicidal attempts (OR = 1.48, 95% CI: 1.16, 1.89). Subgroup analyses indicated higher risks in specific groups: adolescents from Central China (OR = 1.84, 95% CI: 1.46, 2.32), college students (OR = 2.09, 95% CI: 1.66, 2.62). The risk of suicidal ideation was particularly elevated when depression was not controlled (OR = 1.86, 95% CI: 1.53, 2.25). These findings underscore the need for targeted interventions in vulnerable populations.

**Conclusion:**

This meta-analysis demonstrated significant associations between PIU and suicidal behaviors among Chinese adolescents. The findings emphasize the need for targeted interventions, particularly for adolescents from Central and Western China, college students, and those with untreated depression. Focused strategies are required to mitigate the risks associated with PIU and to effectively address suicidal behaviors in these high-risk populations.

**Systematic review registration:**

https://www.crd.york.ac.uk/prospero/display_record.php?ID=CRD42024577593, identifier CRD42024577593.

## 1 Introduction

Globally, about 703,000 people commit suicide each year, accounting for 1.3% of total deaths. 77% of these occur in low-and middle-income countries, making it the second leading cause of death for 15-29-year-olds (1). By 2040, global suicide deaths are predicted to reach 1.03 million (2). Around 67,000 teenagers worldwide die by suicide each year, with over 88% in low- and middle-income countries (3, 4). In China, suicide is a main cause for teenagers (5), and is rising (6). Furthermore, adolescent suicides often occur in clusters, primarily within schools, making educational institutions critical settings for such incidents (7). Adolescent suicide not only has a profound impact on families but also exerts a negative influence on society. It is estimated that each suicide death affects at least 60 individuals, including family members, friends, and colleagues, increasing their risk of suicidal behavior, mental illness, and physical disorders, and impairing their social functioning (8, 9). Consequently, adolescent suicide has become a significant global public health concern.

The World Health Organization defines suicidal behavior as including suicidal ideation (SI), suicide planning (SP), and suicide attempt (SA) (10). SI refers to the repeated or persistent contemplation of ending one’s life, which may involve identifying a method, formulating a plan, or having an intent to act. SP involves the detailed formulation and design of specific steps toward a suicide attempt. SA refer to the actual execution of behaviors intended to end one’s life (11). Suicidal behavior has been identified as a critical and irreversible stage leading to suicide death, serving as one of the most significant warning indicators of potential suicide risk (12, 13). A survey conducted among 229,129 adolescents in 59 low- and middle-income countries revealed prevalence rates of 16.9% for SI, 17% for SP, and 17% for SA (14). Similarly, a survey of 1,103,309 adolescents in China reported that the rates for SI, SP, and SA were 15.4%, 6.4%, and 3.5%, respectively (15). Studies indicated that an individual’s sensitivity to pain, injury, fear, and death was reduced by repeated suicidal behavior, thereby ultimately increasing the risk of suicide (13). Thus, assessing the factors influencing suicidal behavior is crucial for the prevention of suicide deaths.

As of April 2024, the global internet user base has reached 5.44 billion. Of these, approximately 2.93 billion users are located in Asia, accounting for 53.9% of the worldwide total (16). Notably, China, home to the world’s largest internet user base, had 1.092 billion users by December 2023, as reported by the China Internet Network Information Center (CINIC) in its 53rd statistical report on internet development. Among these, 161 million were adolescents, comprising 14.7% of the total (17). Despite the advantages offered by online access for adolescents in education and daily routines, unchecked and excessive engagement was associated with the development of “Problematic Internet Use (PIU)” (18–20). PIU refers to a pattern of excessive or inappropriate internet use that manifests as frequent and prolonged usage, leading to significant negative impacts on an individual’s daily life, social relationships, academic or occupational performance (21). Although PIU can occur at any age, adolescence is considered the most vulnerable period. Adolescents are not only quick to adopt and become engrossed in the latest technologies and social media tools (22), but they are also more susceptible to the potential negative effects of internet use compared to other groups (23). Research indicates that PIU can lead to a range of serious issues among adolescents, including sleep disturbances, musculoskeletal and neurological problems, academic decline, procrastination, impulsivity, reduced self-esteem, diminished social interaction, feelings of loneliness, and suicidal behavior (24).

The prevalence of PIU and suicidal behavior was found to be high among Chinese adolescents, making it essential to explore their association to better understand the mental health status of this population. Studies indicated that cultural differences might have played a significant role in the relationship between mental health issues and behavioral problems (25). The relationship between PIU and suicidal behavior was particularly complex across different cultural contexts, potentially influencing both the manifestation and severity of suicide risk (26–28). According to the Interpersonal-Psychological Theory of Suicide proposed by Thomas Joiner, suicidal behavior primarily stems from three psychological factors: perceived burdensomeness, lack of belongingness, and acquired capability for suicide (29). Chinese adolescents, due to academic pressure and familial expectations, often experienced a sense of being a burden to others. When they were unable to obtain a sense of belonging in real life, they tended to rely on online social interactions as a form of substitute support. However, excessive dependence on the internet may have worsened feelings of loneliness and emotional distress. Furthermore, exposure to suicide-related information online was likely to reduce their fear of death, thereby further increasing the risk of suicidal behavior (30, 31). Although PIU had been identified as a potential risk factor for suicidal behavior, the relationship was not always clear (32). For instance, a study by Yang et al. found that after controlling for depressive symptoms, the association between PIU and suicidal behavior among adolescents was no longer significant, suggesting the complexity of this relationship (33). Therefore, investigating how PIU influences suicidal behavior among Chinese adolescents is crucial for developing effective prevention strategies.

To date, seven systematic reviews have extensively examined this association, four of which are narrative reviews (34–37). While these reviews supported the existence of a connection, the specifics and intensity of this relationship were still uncertain, largely due to the methodological limitations in the reviews. In a meta-analysis spanning diverse regions across Europe and Asia, it was found that PIU remained linked to suicidal behavior even after accounting for confounding variables like depression (38). However, this analysis did not thoroughly explore the relevant influencing factors specific to the adolescent population. Additionally, two recent systematic reviews (39, 40) highlighted the association between adolescent PIU and suicidal behavior, which was particularly pronounced. The reviews were limited by their small scope, each relying on only 3 or 4 studies, and potential influencing factors were not thoroughly examined. Therefore, this study aimed to synthesize previous research on the relationship between PIU and suicidal behavior among Chinese adolescents, with a particular focus on key variables that had not been thoroughly examined in prior studies. These variables included sociodemographic factors (such as age, gender, and region), differences in measurement tools used in research methods, and the control of confounding factors like depression (38). This approach was intended to address the gaps in existing research and lay the foundation for developing more specific intervention strategies in the future. Thus, the present study further explored the relationship between PIU and suicidal behavior among Chinese adolescents through the following research questions (1): What is the strength of the association between PIU and suicidal behavior among Chinese adolescents? (2) Do the control of variables in research methods (including demographic characteristics and factors such as depression) moderate the association between PIU and suicidal behavior?

## 2 Methods

### 2.1 Materials and methods

The PRISMA guidelines for systematic reviews and meta-analyses were adhered to in the conduct of this study (41). The research protocol was officially registered in PROSPERO under the registration number CRD42024577593. It was noted that the PRISMA guidelines, recognized internationally as a standard, were provided as a comprehensive and rigorous framework for systematic reviews and meta-analyses. Meanwhile, the PROSPERO registration platform was utilized to ensure the transparency, reproducibility, and scientific integrity of the research protocol. This platform was used to help reduce research bias and to enable other researchers to track and evaluate the progress and quality of the study more effectively. Through PROSPERO registration, the design and methods of this study were clearly documented and made publicly accessible, thereby laying a solid foundation for subsequent research communication and application.

### 2.2 Search strategy

To explore the relationship between PIU and suicidal behavior, we searched seven databases (PubMed, Web of Science, EMBASE, CNKI, Wanfang Data, VIP Database, and CBM) from their establishment to July 1, 2024. In the search, we used detailed methods. In titles, abstracts, and subject terms, we carefully selected keywords divided into three groups: (“Suicide”, “Problematic Internet Use”, and “Adolescents”). For “Suicide”, we included common and specific terms like “suicide”, “self-harm”, “attempted suicide”, “suicidal behav*”, “suicidal ideation”, etc. For “Problematic Internet Use”, we covered expressions like “excessive internet use”, “internet addiction”, “problematic online behavior”, “compulsive internet use”, “maladaptive internet usage”, etc. For “Adolescents”, we included common and specific terms like “adolescents”, “teenagers”, “young people”, “youth”, “middle school students”, “college students”, etc. To ensure thoroughness, reference lists of the included studies were manually reviewed to identify any additional relevant articles. Additionally, manual cross-referencing of citations was performed to ensure thoroughness. The entire search strategy was meticulously documented to guarantee reproducibility. Further details regarding the search methodology can be found in **Supplementary Material**.

### 2.3 Study selection criteria

Inclusion criteria for the meta-analysis were as follows (1): Population: Studies that focused on Chinese adolescents aged 10 to 24 years (42) (2). Exposure: Research that specifically examined the association between PIU and suicidal behavior (3). Outcomes: Studies that reported relevant outcomes, including SI, SP, SA, with corresponding statistical measures such as Odds Ratio (OR) (4). Study Design: Peer-reviewed articles employing cross-sectional, case-control, or cohort study designs (5). Language: Articles published in either Chinese or English (6). Methodological Rigor: Studies that provided sufficient detail on their methodology, allowing for assessment of bias and overall study quality.

Exclusion criteria were defined as (1): Population: Studies that involved non-Chinese adolescents or included participants outside the age range of 10 to 24 years (2). Exposure: Research that focused on general internet use, other behavioral addictions, or any exposure not directly related to PIU and its association with suicidal behavior (3). Outcomes: Articles that did not report extractable data on suicidal behaviors or provided insufficient statistical information to calculate effect sizes (4). Study Design: Research formats such as reviews, systematic reviews, meta-analyses, conference abstracts, editorials, letters, comments, or case reports were excluded. Studies with a high risk of bias or those lacking peer review were also excluded.

### 2.4 Data extraction

Data extraction was conducted systematically to ensure consistency and accuracy across all included studies. A standardized data extraction form was developed and utilized by two independent reviewers (XBH, CS) to collect essential information, including study characteristics (author(s), year of publication, and study location), population details (sample size, age range, gender distribution, and specific demographic characteristics), exposure variables (definitions and measures of PIU, including criteria, scales, or questionnaires used), and outcome variables related to suicidal behavior (SI, SP, and SA), with corresponding OR and 95% CI where available. Adjustments for potential confounding factors, such as depression, were also noted, and the key findings of each study, particularly those related to the association between PIU and suicidal behavior, were summarized. The initial collection of literature, as well as the identification and removal of duplicate records, was performed using NoteExpress software. To ensure accuracy, all extracted data were cross-checked by a third reviewer (PY), with any discrepancies between the two primary reviewers resolved through discussion and consensus. In cases where consensus could not be reached, a senior researcher (BY) was consulted. The extracted data were then compiled into a comprehensive database for subsequent analysis.

### 2.5 Quality assessment

The quality of the included studies was evaluated using two established tools: the Agency for Healthcare Research and Quality (AHRQ) methodology checklist and the Newcastle-Ottawa Scale (NOS). For cross-sectional studies, the AHRQ tool assessed 11 items, including population definition, sampling method, response rate, variable measurement, and data analysis. Each item was scored as “yes” (1 point), “no” (0 points), or “unclear” (0 points), with a maximum score of 11. Studies scoring 8 or above were rated as high quality, 4 to 7 as moderate quality, and below 4 as low quality. For this meta-analysis, only studies with a score of 4 or higher were included (43). For cohort and case-control studies, the NOS was used, which evaluated studies on three criteria: selection (up to 4 stars), comparability (up to 2 stars), and outcome/exposure ascertainment (up to 3 stars). The NOS scores ranged from 0 to 9 stars, with 7 to 9 stars indicating high quality, 4 to 6 stars moderate quality, and below 4 stars low quality. This analysis included only studies with at least 4 stars to ensure methodological robustness (44). Two reviewers, XBH and SC, independently assessed each study, with any discrepancies resolved through discussion or consultation with a third reviewer, PY. The quality assessment ensured that only reliable, high-quality studies contributed to the meta-analysis.

### 2.6 Statistical analysis

To examine the relationship between PIU and suicidal behavior, including SI, SP, and SA, pooled OR with 95% CI were estimated using random-effects models. STATA software (version 16.0) was employed to conduct all statistical analyses. Heterogeneity across the included studies was assessed through the *I²* statistic and Cochran’s Q test. Significant heterogeneity was considered present when the *I²* statistic exceeded 50% or when Cochran’s Q test yielded a p-value of less than 0.05, warranting the application of a random-effects model (45). To assess the robustness of the overall results, sensitivity analyses were conducted. Specifically, the meta-analysis was re-evaluated by excluding studies with a high risk of bias and by using alternative statistical models (46). Furthermore, Publication bias was initially assessed through funnel plot analysis (47) and Egger’s test (48). If publication bias was detected, the trim-and-fill method was subsequently applied to adjust for it (49). A p-value of less than 0.05 was considered statistically significant. Subgroup analyses were conducted to explore the association between PIU and SI. These analyses were stratified by age groups, measurement tools, geographical regions, control variables including depression, and gender distribution to investigate potential sources of heterogeneity and identify variations in the strength of association across different subgroups. The fixed-effects model was employed to assess differences between these subgroups. All findings were reported in compliance with PRISMA guidelines, ensuring that the analysis was both transparent and reproducible.

## 3 Results

### 3.1 Attributes and quality assessment of the included research

**Figure 1:**
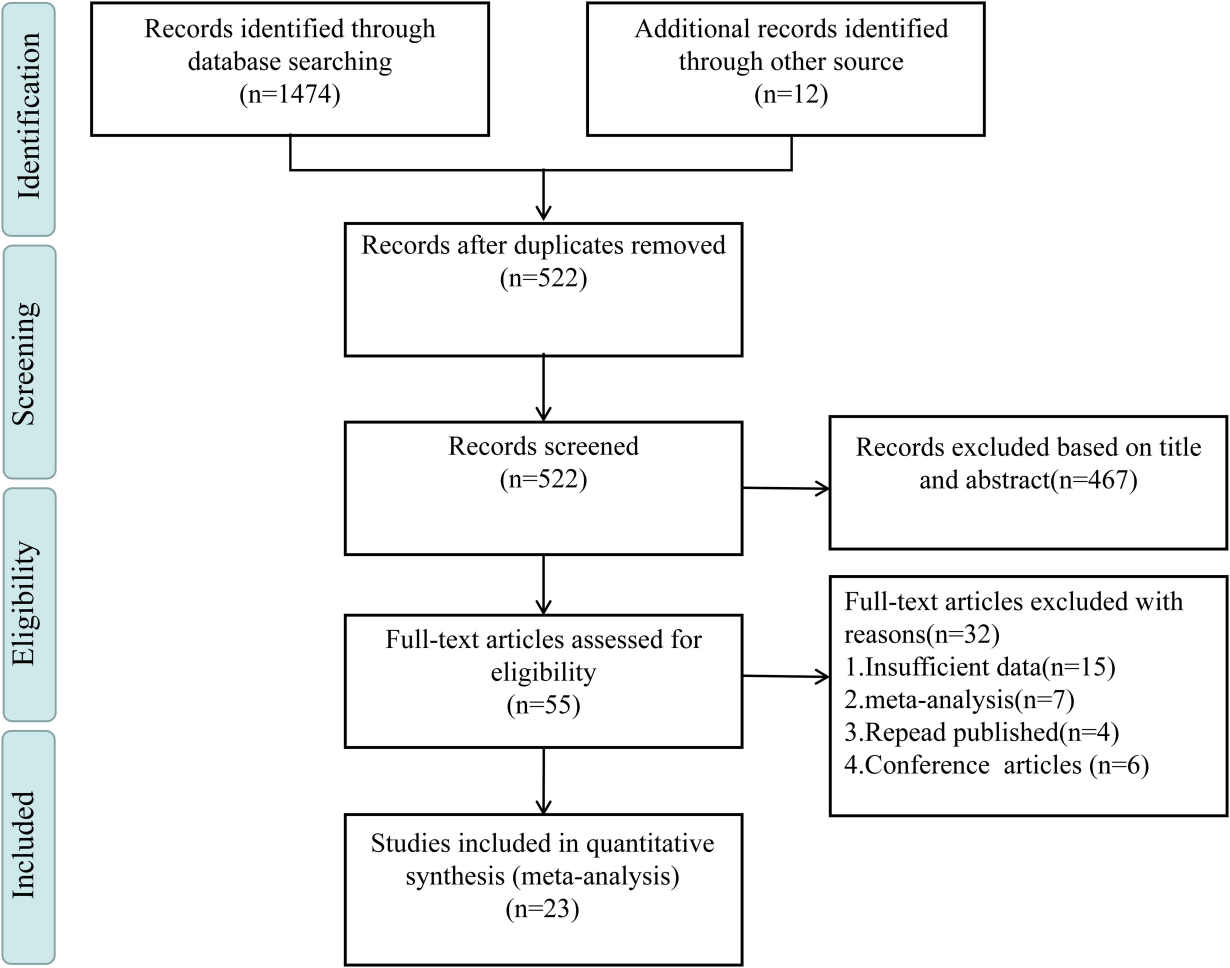
Literature selection process.

According to the predefined search strategy, we initially identified 1,474 relevant articles. After initial screening with NoteExpress, 522 papers remained. Then, after reviewing titles and abstracts, the number reduced to 55. A comprehensive full-text analysis was done, and eventually 23 articles meeting the predefined inclusion criteria (33, 50–71) were selected. All studies were conducted as cross-sectional research. Longitudinal studies were excluded because they were either unavailable within the scope of the search results or did not meet the specific inclusion criteria. These selected studies covered 353,904 participants. Regarding assessment tools for SI, 16 studies (50–57, 59, 61, 63, 65, 68–71) used specialized and validated instruments for measuring SI. For example, they might include professional scales for measuring the frequency, intensity, and duration of suicidal thoughts. Meanwhile, 7 studies (33, 58, 60, 62, 64, 66, 67) used self-designed tools. These might be custom-developed based on specific research questions and populations. In terms of the assessment of PIU, 7 studies (33, 51, 55–57, 59, 64) adopted the Young Internet Addiction Test by Young et al. (72), which might cover multiple dimensions like internet usage time, frequency, behavior patterns, and dependence degree. Additionally, 5 studies (50, 60, 62, 69, 71) chose the Chen Internet Addiction Scale by Chen et al. (73), which might focus on aspects such as loss of control, interference with daily life, and withdrawal reactions. Furthermore, 3 studies (66, 68, 70) used the Mobile Phone Addiction Index translated into Chinese by Liu et al. (74), concentrating on evaluating addiction degree, impact on social and daily life, etc. The remaining 8 studies (52–54, 58, 61, 63, 65, 67) adopted other assessment tools based on specific scenarios and requirements. The sample sizes of the included studies varied significantly. The smallest had 439 participants, while the largest covered 136,266. As detailed in the **Supplementary Materials**, we conducted a very detailed quality assessment. 18 studies were classified as high-quality for excellent performance in research design, methods, data quality, and result reliability. Another 5 met certain standards in various aspects and were categorized as medium-quality. **Table 1** summarizes the main characteristics of these literatures, and **Figure 1** outlines each inclusion stage.

**Table 1:** Characteristics of the included article.

References	Region	Sample size (Male/Female)	Age range /Population	OR (*95%CI*)	Type of assessment (SI)	Type of assessment (PIU)	Adjusted for depression	Study quality scores
Yang et al., 2010 (33)	Center	3507(1818/1689)	15.4 ± 1.6/M	SI: 1.38(0.98, 1.98) SA: 1.24(0.55, 2.81) SP: 1.50(0.96, 2.35)	N/A	IAT	Yes	5
Lin et al. 2014 (50)	Eastern	9510(4593/4917)	14.69 ± 1.74/M	SI: 1.25(1.08, 1.44) SA: 1.59(1.29, 1.96)	KIDDI-SADS-E	CIAS	Yes	8
Zhang et al., 2014 (51)	Center	3620(212/3408)	18.03 ± 1.93/C	SI: 1.54(1.07, 2.23)	SIOSS	IAT	Yes	7
Wang et al., 2014 (52)	Eastern	5051(2179/2872)	M	SI: 1.28(1.00, 1.62) SA: 1.28(1.91, 2.55)	KIDDI-SADS-E	PCPU-Q	Yes	8
Zhang et al., 2018 (53)	Western	2360(1071/1289)	15.03 ± 1.76/M	SI: 2.47(1.69, 3.62)	CAHRBQ	CAHRBQ	No	7
Wang et al., 2019 (54)	Western	26688(13004/13684)	16.8 ± 1.60/M	SI: 1.15(1.05, 1.27)	SCL-90-R	SCL-90-R	Yes	11
Lu et al. 2020 (55)	Western	5864(2093/3657)	19.9 ± 1.52/C	SI: 1.87(1.61, 2.17)	BSS	IAT	No	9
Kuang et al., 2020 (56)	Western	136266(60047/76219)	18.6 ± 1.90/M and C	SI: 1.19(1.14, 1.23)	BSS	IAT	No	10
Guo et al., 2020 (57)	Western	31659(16109/15550)	C	SI: 2.88(2.74, 3.03) SA: 2.29(1.85, 2.83) SP: 2.56(2.32, 2.82)	SBQ-R	IAT	Yes	10
Yu et al., 2020 (58)	Eastern	1066(602/464)	M	SI: 3.09(2.10, 4.54)	N/A	DSM-V	No	10
Huang et al., 2020 (59)	Western	12507(6067/6440)	16.6 ± 0.80/M	SI: 1.41(1.28, 1.56) Male: 3.19(2.84, 3.58) Female: 3.58(3.16, 4.06)	SBQ-R	IAT	Yes	10
Shen et al., 2020 (60)	Center	8098(3592/4506)	C	SI: 2.27(1.84, 2.78) SA: 1.67(1.26, 2.22) SP: 1.04(0.73, 1.47)	N/A	CIAS	No	10
Pan et al., 2020 (61)	Eastern	998(547/451)	M	SI: 3.20(1.54, 6.61)	CAHRBQ	CAHRBQ	No	8
Shen et al., 2020 (62)	Center	627(259/368)	C	SI: 1.94(1.30, 2.91) SA: 1.20(0.68, 2.11) SP: 0.76(0.38, 1.52)	N/A	CIAS	No	10
Wen et al., 2021 (63)	Eastern	984(529/455)	14.98 ± 1.61/M	SI: 1.81(1.08, 3.03)	CAHRBQ	CAHRBQ	No	7
Chang et al., 2021 (64)	multiple regions	16130(8371/7759)	15.22 ± 1.79/M	SI: 1.67(1.48, 1.90) SA: 2.63(2.22, 3.11) SP: 2.36(2.02, 2.75)	N/A	IAT	No	10
Wang et al., 2022 (65)	Western	2380(1281/1099)	M	SI: 1.94(1.37, 2.74)	CAHRBQ	CAHRBQ	No	7
Huang et al., 2022 (66)	Center	439(58/381)	18.8 ± 1.70/C	SI: 2.60(1.13, 6.01)	N/A	MPAI	No	10
Junus et al., 2023 (67)	Eastern	3430(2573/857)	19.44 ± 5.36/M and C	SI: 0.97(0.91, 1.04) SA: 0.92(0.77, 1.10)	N/A	IGDS9-SF	No	10
Wang et al., 2023 (68)	Western	18723(6531/12192)	C	SI: 1.70(1.53, 1.88) Male: 1.70(1.42, 2.03) Female: 1.69(1.49, 1.91) SA: 1.48(1.18, 1.86)	SIOSS	MPAI	Yes	10
Kang et al., 2023 (69)	multiple regions	5366(1846/3520)	20.02 ± 1.38/C	SI: 2.34(1.84, 2.98)	SIOSS	CIAS	Yes	10
Cheng et al., 2024 (70)	multiple regions	18900(9416/9484)	14.99 ± 1.64/M and C	SI: 1.22(1.09, 1.37) SA: 1.06(0.90, 1.24) SP:1.18(1.04, 1.34)	CAHRBQ	MPAI	Yes	10
zhang et al., 2024 (71)	Eastern	39731(21614/18117)	13.49 ± 0.76/M	SI: 1.93(1.80, 2.10) Male: 1.95(1.75, 2.19) Female: 1.91(1.69, 2.14)	PHQ-9	CIAS	No	10

C, Cross-sectional studies; C, College; M, Middle school; SI, Suicidal Ideation; SP, Suicidal Plan; SA, Suicide Attempt; CAHRBQ, China Adolescents Health-related Behavior Questionnaire; N/A, Self-designed Scale; KIDDI-SADS-E, Schedule for Affective Disorders and Schizophrenia for School-Age Children - Epidemiologic Version; SIOSS, The Self-rating Idea of Suicide Scale; PCPU-Q, Problematic Cellular Phone Use Questionnaire; SCL-90-R, Symptom Checklist-90-Revised; BSS, Beck Scale for Suicide Ideation; SBQ-R, Suicidal Behaviors Questionnaire-Revised; PHQ-9, Patient Health Questionnaire-9; IAT, Internet Addiction Test designed by Young; CIAS, Chinese Internet Addiction Scale; MPAI, Mobile Phone Addiction Index.

### 3.2 Homogeneity test and meta-analysis

#### 3.2.1 A homogeneity test and meta-analysis assessed the PIU-SI link

**Figure 2:**
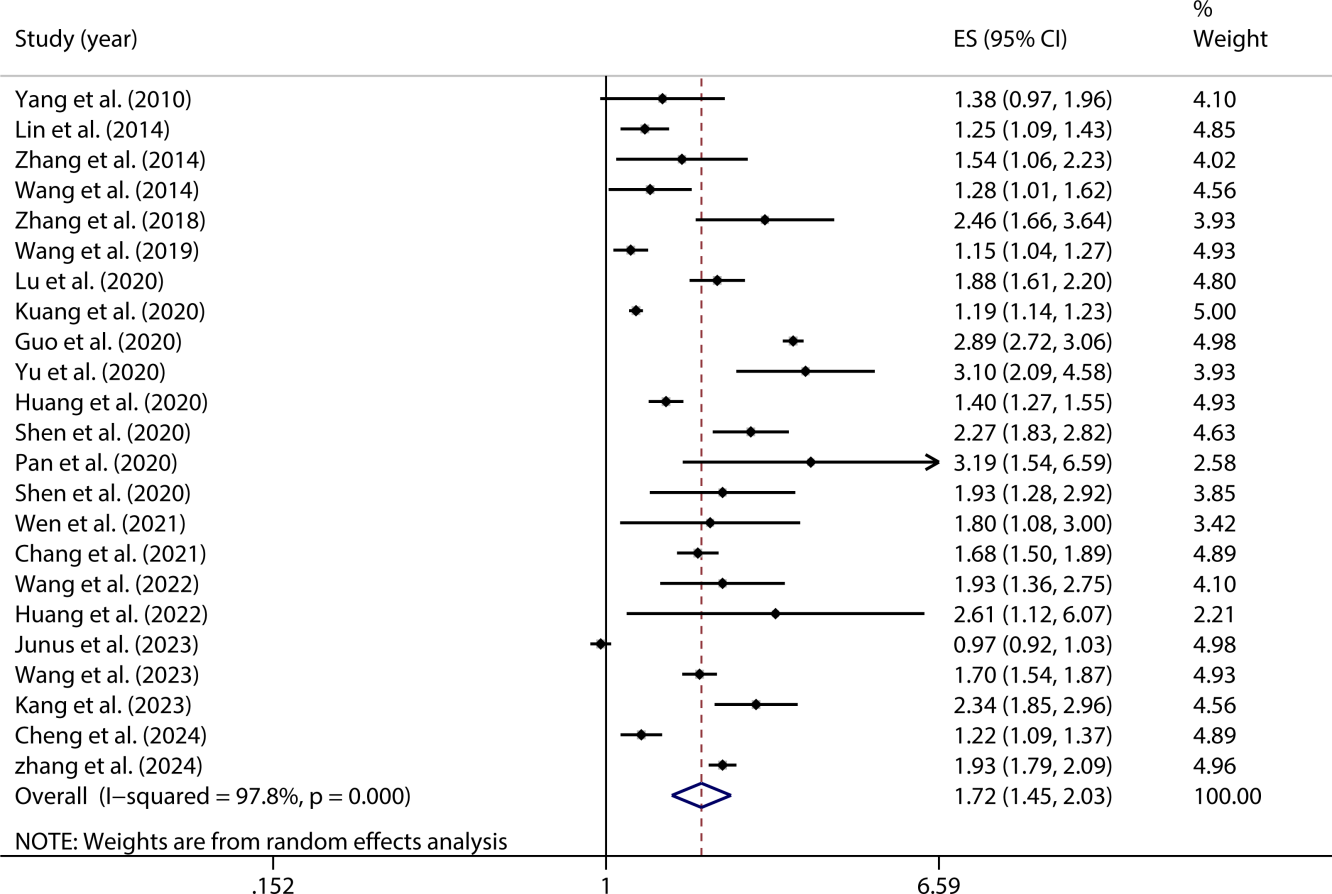
Forest plot of the relationship between PIU and SI.

A homogeneity test conducted on the 23 studies included in this analysis indicated substantial heterogeneity (*I²* = 97.8%, *P* < 0.001). Given this considerable level of heterogeneity, a random-effects model was utilized to estimate the overall effect. The model produced a pooled effect size of (OR = 1.72, 95% CI: 1.45, 2.03), suggesting that individuals with PIU were 1.72 times more likely to experience SI compared to those without PIU. This association was found to be statistically significant (*Z* = 6.33, *P* < 0.001), as shown in **Figure 2**.

#### 3.2.2 Managing the heterogeneity in the PIU-SI relationship

During the analysis, significant heterogeneity was uncovered through the application of the random-effects model among the studies included in the Meta-analysis. This heterogeneity, which indicated substantial variability in the results across different studies, was recognized as a challenge to the consistency and interpretability of the overall findings. Given the potential impact of this variability on the accuracy of the Meta-analysis, it was deemed essential that additional analyses be undertaken to better understand and address the sources of this heterogeneity. In response, a subgroup analysis was performed, wherein the studies were divided into distinct categories based on specific predefined characteristics, such as population demographics. By isolating and evaluating the factors contributing to the observed heterogeneity, this approach allowed for a more nuanced interpretation of the data. The results of the subgroup analysis provided deeper insights into the variability among studies, thereby enhancing the robustness and validity of the overall conclusions drawn from the Meta-analysis.

#### 3.2.3 Comparison of PIU and SI through subgroup analysis

A subgroup analysis was undertaken to explore various elements linked to both PIU and SI. These elements included geographical region, age, gender, the specific instruments utilized to assess PIU and SI, as well as the consideration of depression adjustments, as presented in **Table 2**.

**Table 2:** Subgroup analysis showing OR of SI for PIU.

Variables	95% Cl for OR		Heterogeneity test		Heterogeneity between the groups (*P*-value)
	*OR*	*95% Cl*	*I^2^* (%)	*P*-value	
**Region**					< 0.001
Eastern(n=7)	1.67	1.20, 2.33	97.30	< 0.001	
Western(n=8)	1.72	1.26, 2.35	98.90	< 0.001	
Center(n=5)	1.84	1.46, 2.32	47.40	0.107	
multiple regions(n=3)	1.66	1.21, 2.29	93.10	< 0.001	
**Age**					< 0.001
Middle school student(n=12)	1.66	1.42, 1.94	89.80	< 0.001	
College student(n=8)	2.09	1.66, 2.62	93.10	< 0.001	
Middle school and College students(n=3)	1.12	0.96, 1.30	93.90	< 0.001	
**PIU measures**					< 0.001
IAT(n=7)	1.65	1.15, 2.36	99.00	< 0.001	
CIAS(n=5)	1.88	1.47, 2.40	90.00	< 0.001	
MPAI(n=3)	1.54	1.13, 2.09	89.80	< 0.001	
Other measures tool(n=8)	1.66	1.31, 2.12	91.70	< 0.001	
**SI measures**					< 0.001
Unvalidated measures tool(n=7)	1.80	1.26, 2.58	95.90	< 0.001	
Validated measures tool(n=16)	1.69	1.39, 2.06	98.00	< 0.001	
**Adjusted for depression**					< 0.001
Yes(n=10)	1.55	1.18, 2.03	97.90	< 0.001	
No(n=13)	1.85	1.53, 2.25	96.30	< 0.001	
**Sex**					0.54
Male(n=3)	2.20	1.51, 3.22	95.90	< 0.001	
Female(n=3)	2.26	1.43, 3.55	97.80	< 0.001	

IAT, Internet Addiction Test designed by Young; CIAS, Chinese Internet Addiction Scale; MPAI, Mobile Phone Addiction Index.

**Figure 3:**
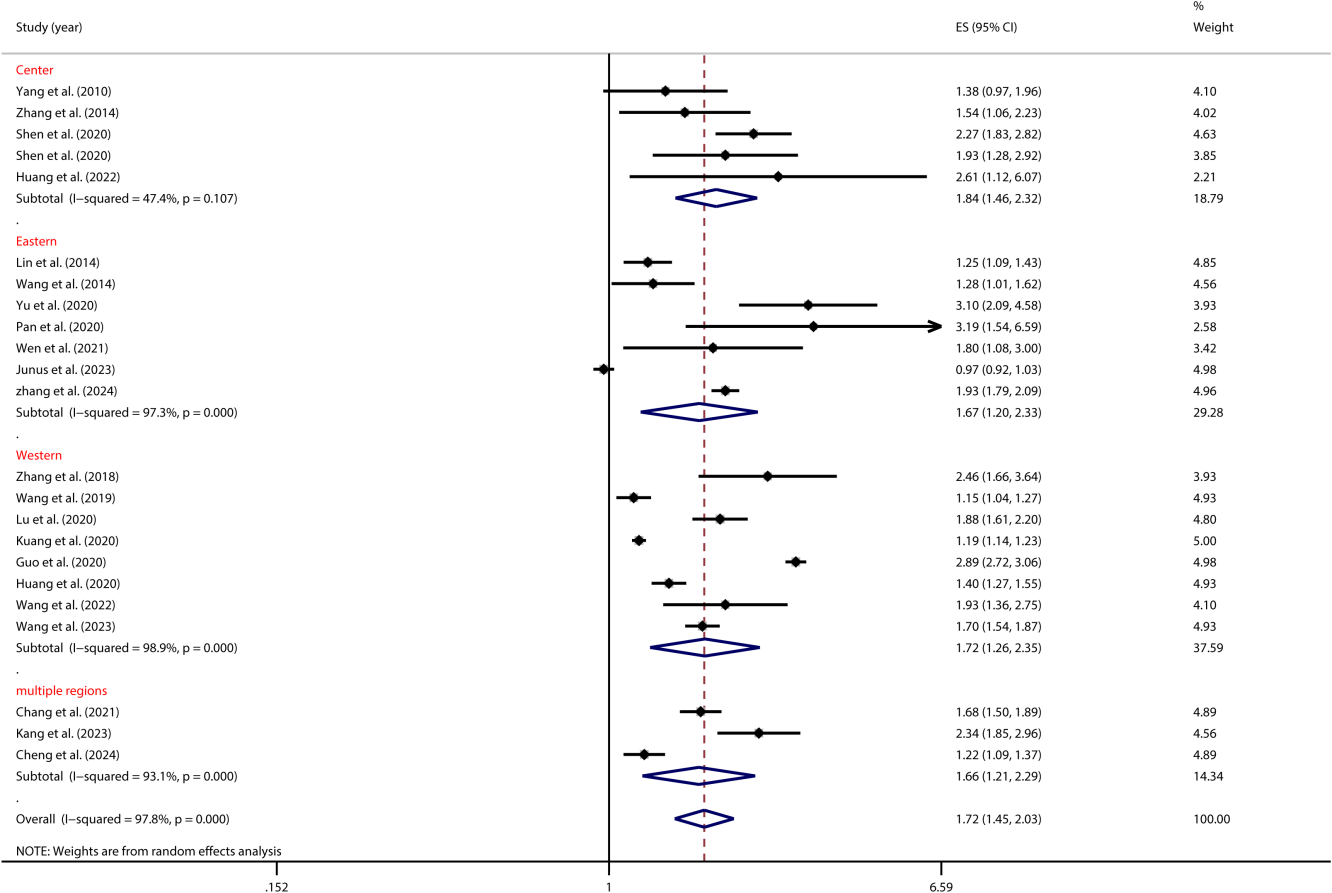
Moderation effect of regional differences on PIU and SI.

As shown in **Figure 3**, substantial regional differences were identified as moderators in the association between PIU and SI, as outlined in **Table 2**. Adolescents from the Central region exhibited the highest vulnerability to PIU in the context of SI (OR = 1.84, 95% CI: 1.46, 2.32). On the other hand, those from the Eastern region displayed a significantly lower risk of SI (OR = 1.67, 95% CI: 1.20, 2.33).

**Figure 4:**
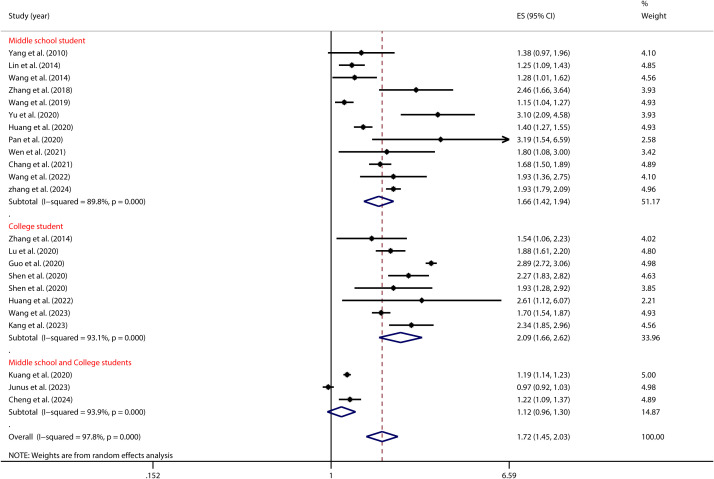
Moderation effect of age on PIU and SI.

As depicted in **Figure 4**, age significantly influenced the relationship between PIU and SI, as shown in **Table 2**. Specifically, Chinese college students with PIU had a higher risk of SI (OR = 2.09, 95% CI: 1.66, 2.62) than middle school students (OR = 1.66, 95% CI: 1.42, 1.94).

**Figure 5:**
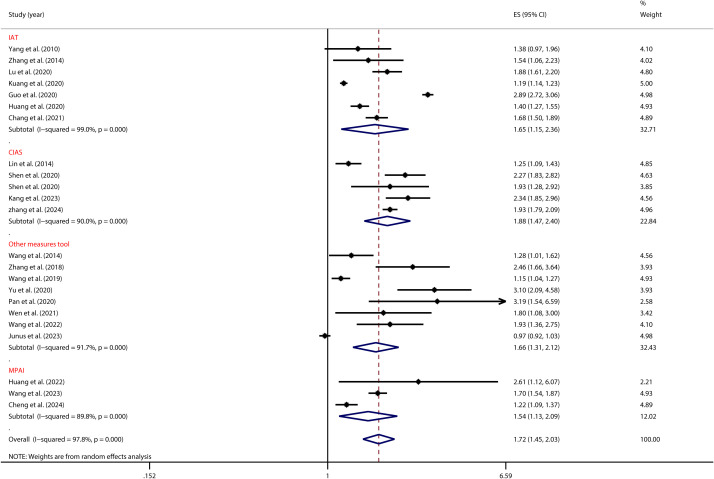
Moderation effect of PIU measures on PIU and SI.

**Figure 6:**
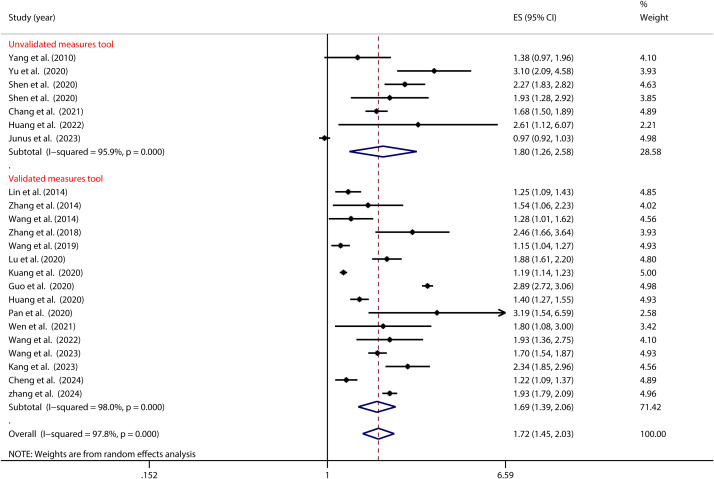
Moderation effect of SI measures on PIU and SI.

As illustrated in **Figure 5**, the tool used to measure PIU significantly moderated the relationship between adolescent PIU and SI (see **Table 2**). Specifically, adolescents with mobile phone addiction exhibited a lower risk of SI when assessed using the MPAS (OR = 1.54, 95% CI: 1.13, 2.09) compared to those evaluated with the CIAS (OR = 1.88, 95% CI: 1.47, 2.40). Likewise, **Figure 6** illustrates that the tool used to measure SI significantly moderated the relationship between adolescent PIU and SI (see **Table 2**). The results indicated that among adolescents with PIU, the use of a validated SI measurement tool (OR = 1.69, 95% CI: 1.39, 2.06) yielded a lower risk compared to an unvalidated tool (OR = 1.80, 95% CI: 1.26, 2.58).

**Figure 7:**
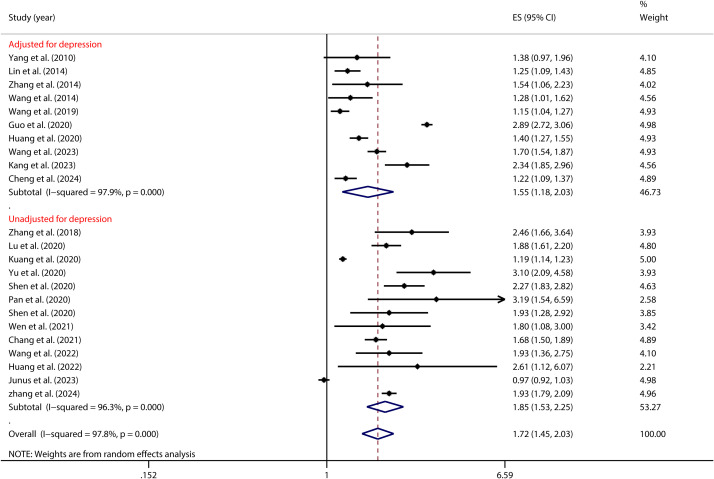
Moderation effect of depression control on PIU and SI.

Furthermore, as illustrated in **Figure 7**, the control of depression significantly moderated the association between PIU and SI (as detailed in **Table 2**). Notably, among Chinese adolescents, the risk of SI was highest when depression was not managed (OR = 1.85, 95% CI: 1.53, 2.25). In contrast, when depression was effectively managed, the risk substantially decreased (OR = 1.55, 95% CI: 1.18, 2.03).

### 3.3 A homogeneity test and meta-analysis assessed the PIU-SP link

**Figure 8:**
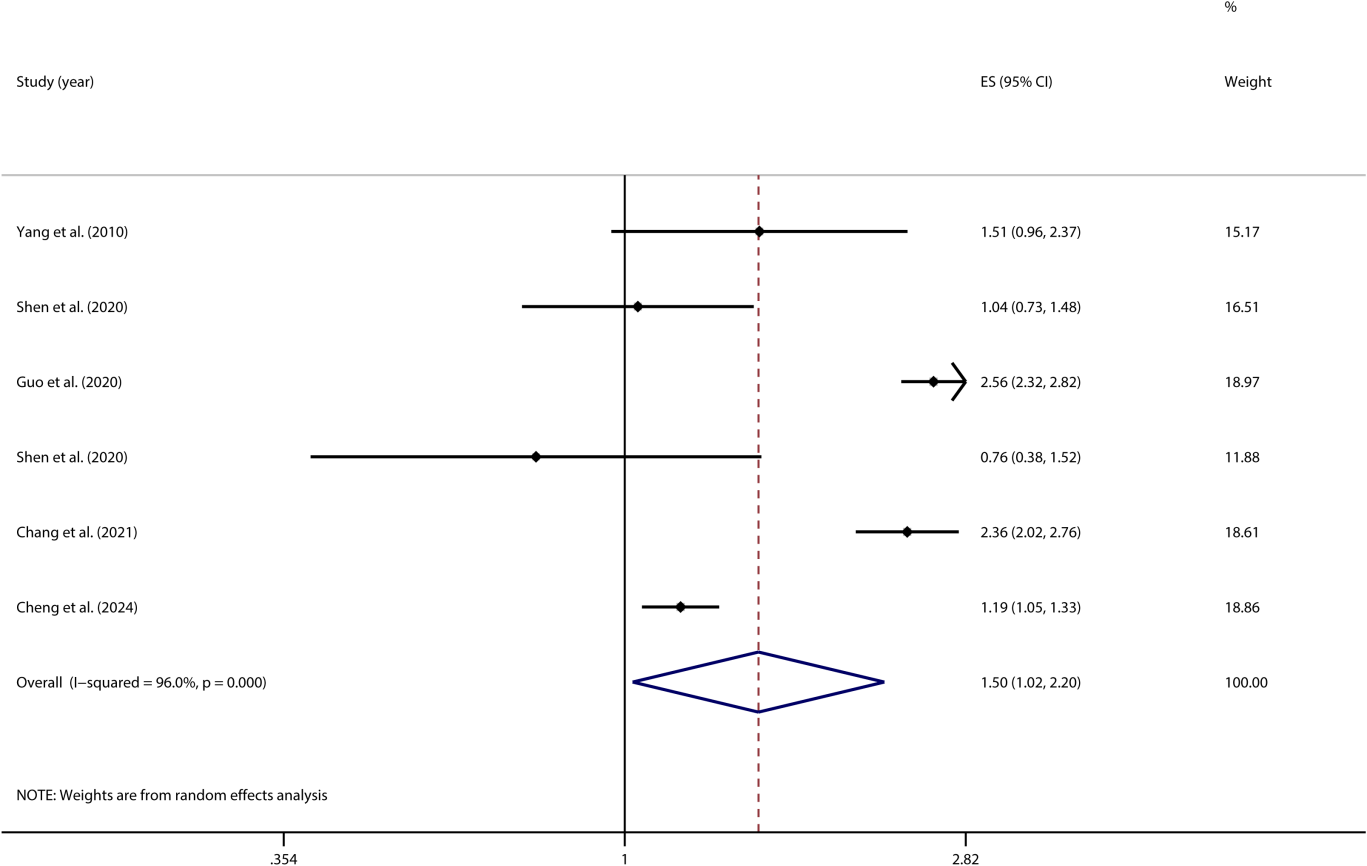
Forest plot of the relationship between PIU and SP.

A homogeneity test conducted on 6 independent samples examining the link between PIU and SP revealed significant heterogeneity across the studies (*I²* = 96.0%, *p* < 0.001). Given this high level of variability, a random-effects model was employed to estimate the overall effect. The model produced a pooled effect size of OR = (1.50, 95% CI: 1.02, 2.20), indicating that individuals with PIU were 1.50 times more likely to engage in suicidal planning compared to those without PIU. This association was statistically significant (*Z* = 2.08, *P* = 0.037) (see **Figure 8**).

### 3.4 A homogeneity test and meta-analysis assessed the PIU-SA link

**Figure 9:**
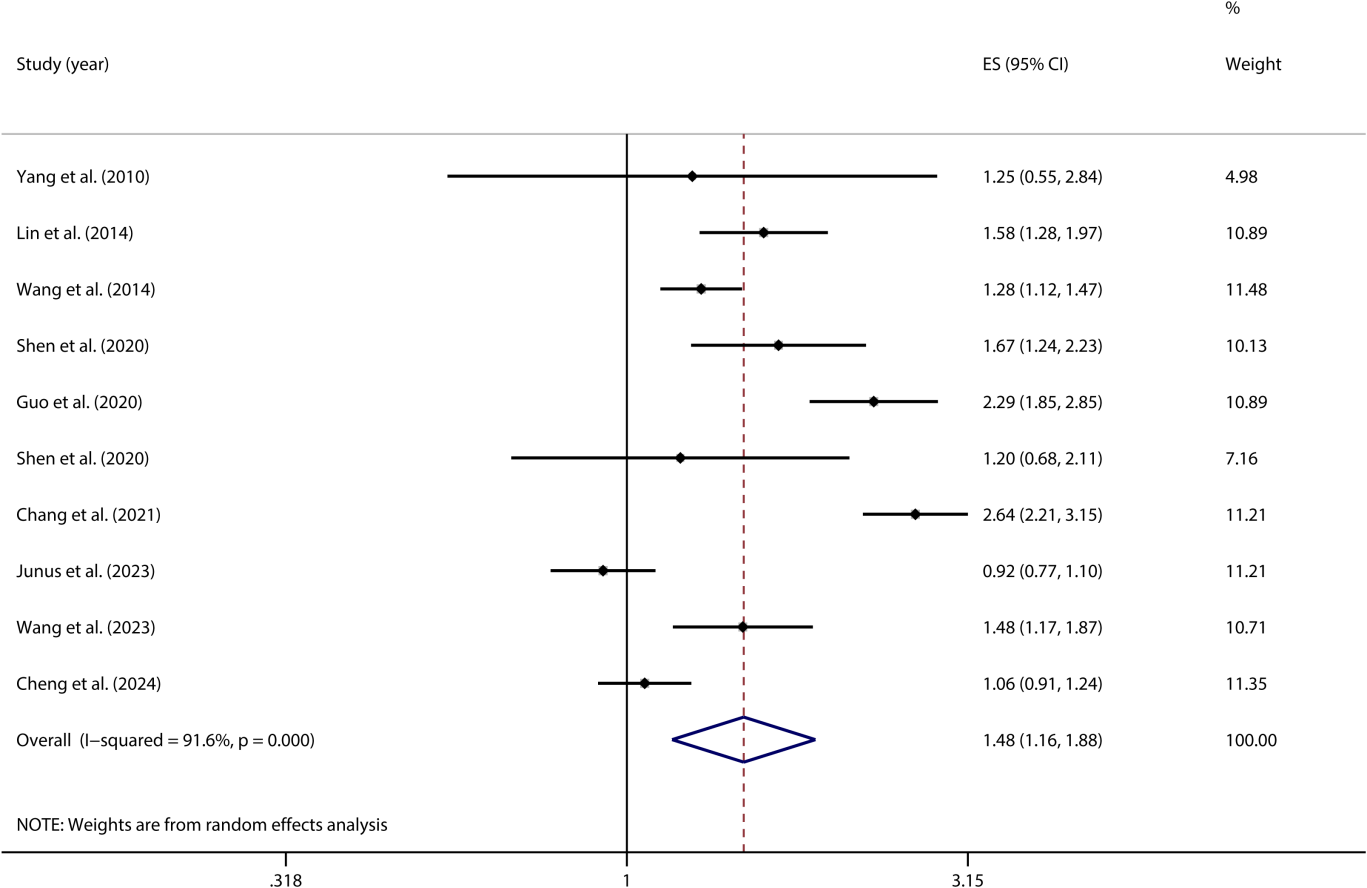
Forest plot of the relationship between PIU and SA.

A homogeneity test conducted on 10 independent samples examining the link between PIU and SA indicated substantial heterogeneity across the studies (*I²* = 91.6%; *p* < 0.001). Given this considerable level of heterogeneity, a random-effects model was utilized to provide a more accurate estimation of the overall effect. The model produced a pooled effect size of OR = 1.48 (95% CI: 1.16, 1.89), indicating that individuals with PIU were 1.48 times more likely to attempt suicide compared to those without PIU. This association was statistically significant (*Z* = 3.20, *P* = 0.001) (see **Figure 9**).

### 3.5 Sensitivity analysis

**Figure 10:**
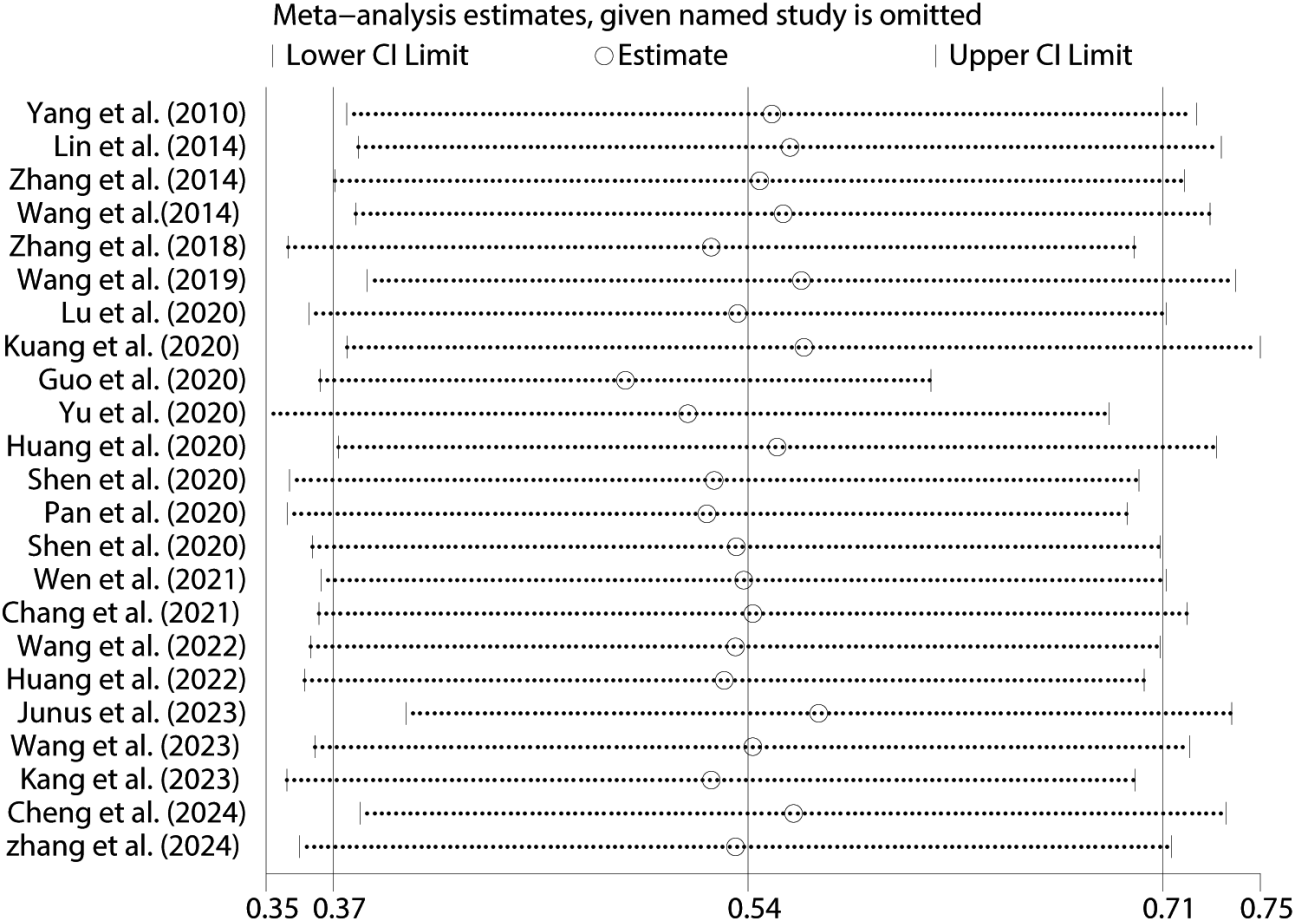
Sensitivity analysis between PIU and SI.

To evaluate the robustness of the research findings, sensitivity analyses were conducted on the correlations between PIU and SI (see **Figure 10**), SP (see **Supplementary Materials**), and SA (see **Supplementary Materials**). The results indicated that the overall correlation coefficients exhibited only minor variations, thereby highlighting the stability and reliability of these findings.

### 3.6 Publication bias

**Figure 11:**
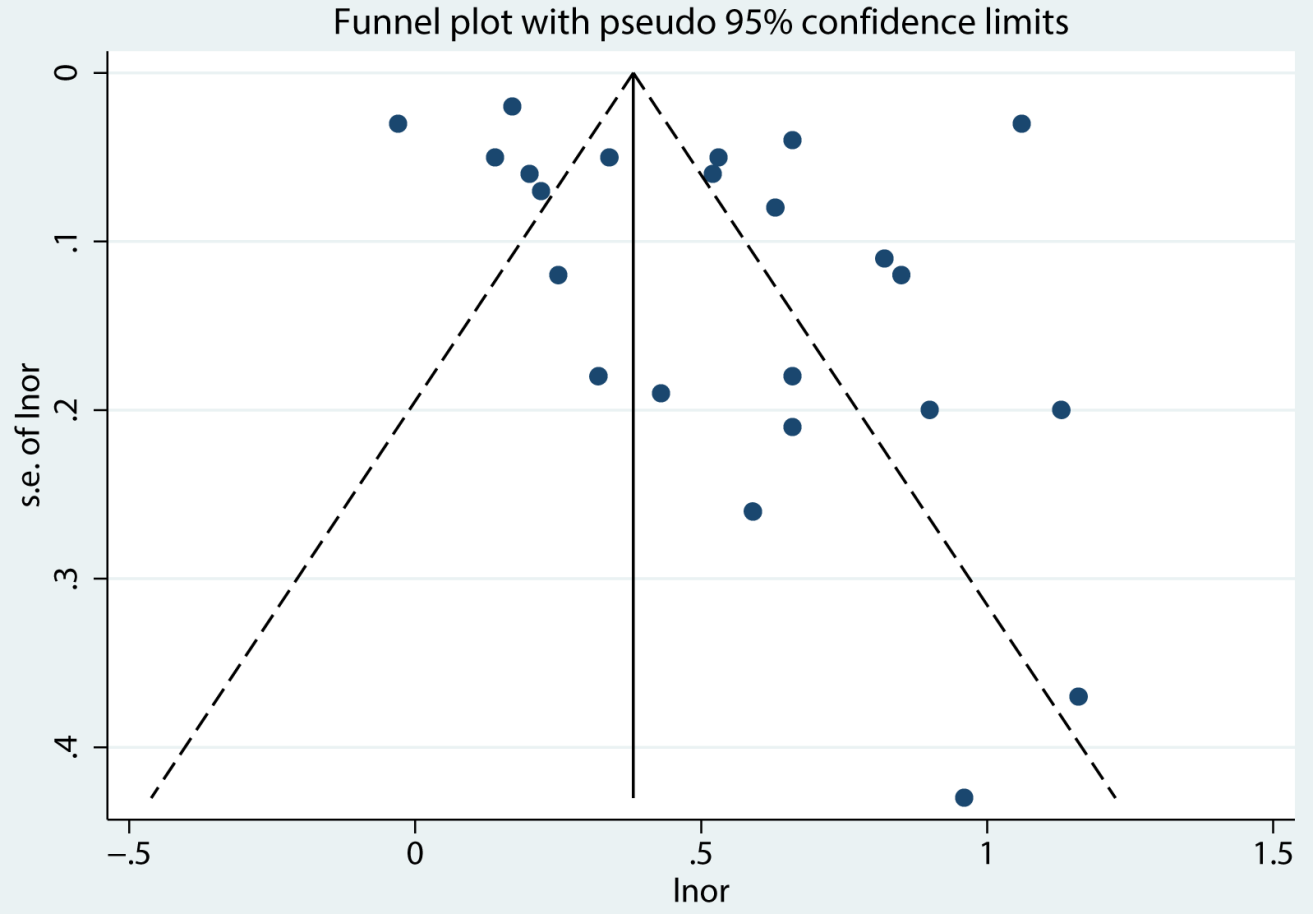
Funnel plot between PIU and SI.

**Figure 12:**
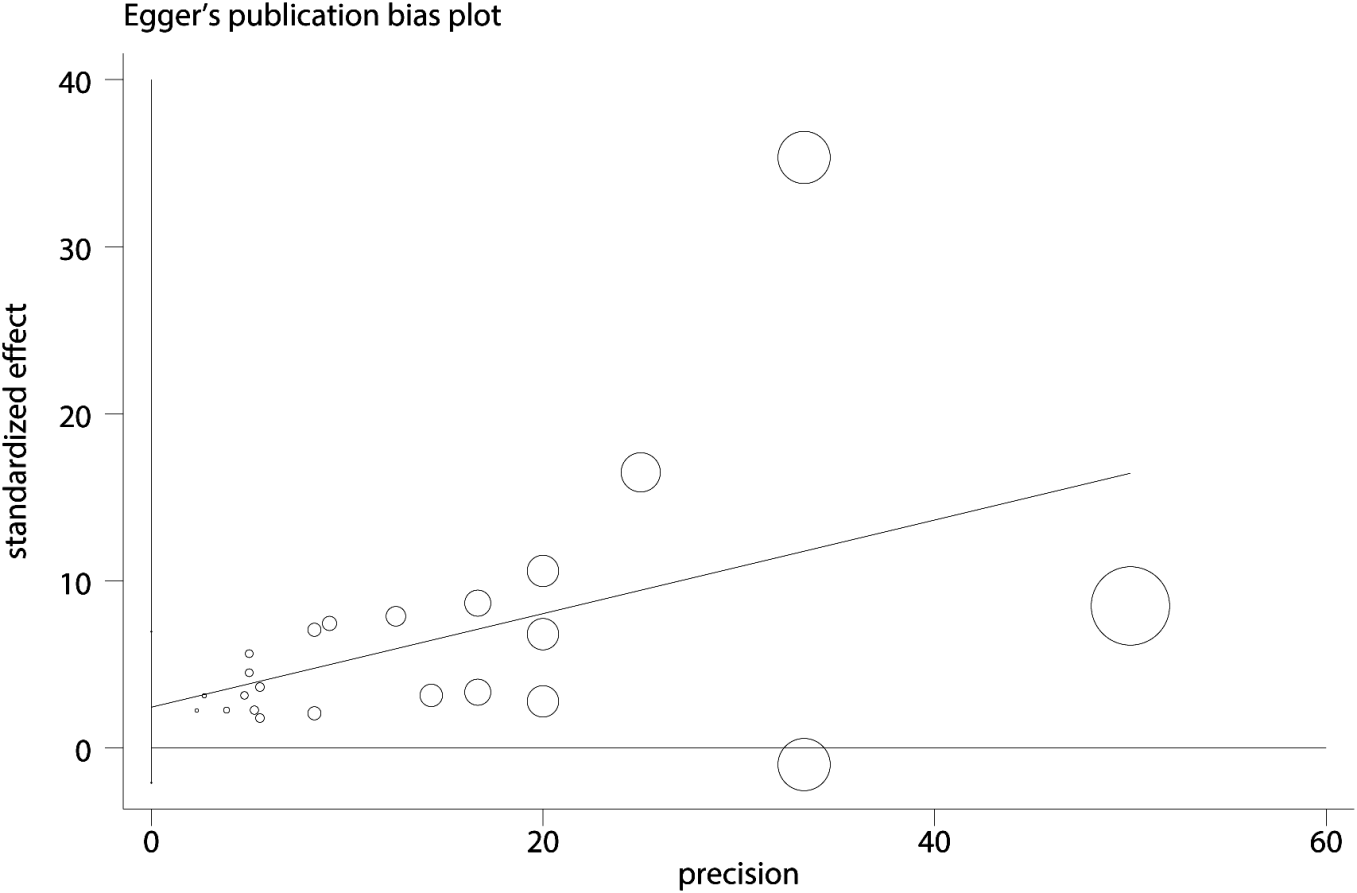
Egger plot between PIU and SI.

The correlations between PIU and SI (**Figure 11**), SP (see **Supplementary Materials**), and SA (see **Supplementary Materials**) were evaluated using funnel plots. The plots demonstrated a balanced distribution on both sides. Additionally, Egger’s regression tests were conducted for PIU and SI (*t* = 1.12, *p* = 0.95) (**Figure 12**), SP (*t* = 0.37, *p* = 0.72) (see **Supplementary Materials**), and SA (*t* = 0.83, *p* = 0.45) (see **Supplementary Materials**). None of these tests revealed statistically significant differences, further indicating that no substantial publication bias was present.

## 4 Discussion

In recent years, adolescent suicidal behavior has become a global public health challenge (75). Meanwhile, PIU has drawn more attention as a factor for adolescent problem behaviors (76). Despite research, a clear consensus on the relationship between PIU and suicidal behavior has remained elusive (77). This meta-analysis suggested that Chinese adolescents with PIU had 1.72 times higher odds of having SI, 1.50 times higher odds of experiencing SP, and 1.48 times higher odds of engaging in SA. In contrast, another meta-analysis covering multiple countries in Europe and Asia found that the risks of SI, SP, and SA increased by 2.95 times, 3.17 times, and 2.81 times, respectively (38). This suggested that the risk of suicidal behavior due to PIU among Chinese adolescents might have been relatively lower. The difference could be attributed to the influence of different cultures, environments, and mechanisms. Nevertheless, the association between PIU and suicidal behavior among Chinese adolescents was found to be consistent with Wang et al.’s conclusion (39). This phenomenon could be attributed to: Firstly, a complex interaction was likely to exist between PIU and suicidal behavior, in which the internet served as a platform exposing adolescents to suicide-related information and peers, thereby increasing the risk of imitation and participation in suicidal behaviors (78). This risk was further amplified in the anonymous online environment, where adolescents could explore suicidal thoughts without fear of judgment and obtain related information through pro-suicide websites (79). Anonymity not only potentially led to deindividuation, reducing self-awareness, but also heightened sensitivity to self-harm (50, 80). Furthermore, adolescence was a critical period for identity formation, during which adolescents might have found online groups with similar mindsets. These groups might have sought validation by encouraging suicidal behaviors, further alienating individuals from mainstream culture (50, 81). Particularly for those vulnerable to social rejection and harm, PIU might have become an unhealthy coping mechanism, exposing them to additional online risks, such as the dissemination of pornographic content and cyberbullying, thereby exacerbating feelings of isolation and psychological stress (35, 82, 83). This process was further intensified by social reinforcement mechanisms, significantly increasing the risk of suicidal behavior. Secondly, PIU was identified as addictive. According to the I-PACE model, addictive behaviors were understood to result from complex interactions among vulnerability factors, emotional and cognitive responses, and executive functions (84–86). Prolonged PIU might have caused changes in the brain structure and function of adolescents, especially in regions related to executive control and decision-making. For instance, excessive use was observed to lead to a decline in prefrontal cortex function, affecting judgment and decision-making, thereby increasing suicide risk (87–89). Moreover, PIU might have led to neuroadaptive changes, distorting the perception of rewards and punishments, and increasing the propensity for suicidal behavior (90, 91). This interacted with the social isolation caused by PIU, weakening the ability to cope with emotions, and prompting the transformation of internal distress into external experiences, ultimately triggering suicidal behavior (92, 93). Additionally, PIU was noted to cause depersonalization, damage social functions, and lead to impulse control disorders, thereby increasing the incidence of suicidal behavior (94). Finally, a strong correlation was observed between PIU and mental health issues such as anxiety and depression, both of which have been identified as significant predictors of suicide. While PIU may have initially served as a coping strategy, its prolonged use could have worsened these mental health problems, ultimately elevating the risk of suicide (95). In summary, PIU was determined to have a direct impact on adolescent mental health, contributing to an increased suicide risk through intricate interaction processes.

### 4.1 The moderating role of region

The findings of this study revealed a significant moderating effect of regional differences on the relationship between PIU and SI among adolescents. Specifically, adolescents in the central and western regions of China with PIU faced a significantly higher risk of SI, whereas those in the eastern regions were at a lower risk. Several potential factors might have contributed to these regional differences. Firstly, economic development in China was uneven, with the eastern regions being more developed compared to the central and western regions. This economic disparity influenced various aspects of life, including access to resources that could have mitigated the impact of PIU. As a result, adolescents in economically disadvantaged areas might have experienced higher levels of stress due to poverty and limited opportunities, which could have exacerbated the effects of PIU on SI (96). Moreover, the level of urbanization in the eastern regions provided more social and recreational opportunities, potentially reducing the negative impact of PIU. Additionally, social support systems varied across regions, with the eastern regions potentially offering stronger and more diverse networks that could have helped adolescents cope with the challenges associated with PIU. In contrast, weaker social support structures in the central and western regions might have led to greater isolation and increased vulnerability to SI among adolescents with PIU (97, 98). Furthermore, cultural attitudes towards mental health might have differed, with more traditional views in the less-developed regions possibly leading to stigmatization and reduced help-seeking behavior (99). Educational disparities also played a significant role. It is likely that the eastern regions had better mental health education and awareness programs, which could have helped adolescents and their families recognize and address PIU early on. Conversely, lower levels of mental health education in the central and western regions might have contributed to a lack of awareness about the dangers of PIU, thereby increasing the risk of severe outcomes like SI (100, 101). Lastly, higher digital literacy in the eastern regions may have equipped adolescents with better coping strategies for managing internet use, thereby reducing the likelihood of PIU.

### 4.2 The moderating role of age

Moreover, the analysis demonstrated a notable moderating effect of age on the PIU-SI association, revealing that university students were more vulnerable to SI than middle school students when afflicted by PIU. Several key factors may explain the significant age moderation in the PIU-SI relationship. Firstly, developmental differences between university students and middle school students were considered pivotal. University students, being at a more advanced stage of psychological development, faced heightened levels of academic pressure, career uncertainty, and the challenges associated with independent living (102, 103). These factors were likely to exacerbate the effects of PIU, thereby increasing the risk of SI. In contrast, middle school students were generally in more protected environments, characterized by lower social pressures and less psychological maturity, which may have buffered them against the severe consequences of PIU. Secondly, there was a distinct difference in how these age groups engaged with social networks and experienced loneliness. University students were more inclined to rely on virtual social interactions, which could have intensified feelings of loneliness and isolation—both strongly correlated with SI. Conversely, middle school students typically maintained more in-person social interactions, which may have mitigated the psychological impact of PIU (83). Lastly, the availability and accessibility of mental health services differed significantly between these age groups. University students, often residing away from home, might have experienced limited access to support systems, thereby worsening the mental health issues associated with PIU. On the other hand, middle school students generally had more immediate access to parental and school-based support, which could have mitigated the risk of SI (104). These findings underscored the importance of developing age-specific intervention strategies. Educational institutions played a crucial role in implementing programs tailored to the distinct challenges faced by different age groups. Specifically, school-based interventions for middle school students and college wellness initiatives might have proven particularly effective in addressing the unique challenges these populations encountered in managing internet use and mitigating its potential harms.

### 4.3 The moderating role of depression

Furthermore, this study revealed that controlling for depression significantly moderated the relationship between PIU and SI. This finding suggested that the elevated risk of SI among adolescents experiencing PIU could be partially attributed to underlying depressive symptoms, although other variables also played critical roles. This result was consistent with the findings of Wang et al. (52), who noted that depressive symptoms intensified the negative effects associated with PIU. A plausible explanation for this phenomenon was the close association between depressive disorders and PIU. Adolescents suffering from depression might have resorted to excessive internet use as a means of escaping psychological distress. However, such reliance on the internet often escalated into PIU, which, in turn, could have exacerbated depressive symptoms, creating a self-perpetuating cycle that amplified the risk of SI (28, 105). Moreover, adolescents with depression frequently experienced severe social isolation and a lack of effective coping strategies, which further increased their susceptibility to excessive internet use. While engaging with the online environment might have provided temporary relief, it often led to deeper psychological issues, thereby raising the likelihood of SI (106, 107). Given the complexities involved, it was deemed essential that the risk of SI in adolescents with PIU be assessed, irrespective of the presence of depression. Thus, a multifaceted approach—including comprehensive mental health evaluations, early identification of risk factors, and tailored interventions—was considered crucial for reducing the risk of SI in this vulnerable population.

### 4.4 The moderating role of SI measurement tools

In addition, the type of measurement tool used to assess SI was found to moderate the relationship between PIU and SI. Specifically, the correlation between PIU and SI was observed to be lower when SI was measured using validated tools compared to unvalidated ones. This finding can be plausibly explained by differences in the validity of the measurement instruments. Validated tools have undergone rigorous validation processes, ensuring higher reliability and validity, which allows for a more accurate assessment of SI. In contrast, unvalidated tools may have introduced measurement errors or biases, potentially inflating the observed correlations between PIU and SI (108). Consequently, when validated tools were employed, the positive correlation between PIU and SI was more likely to reflect the true relationship, rather than being exaggerated by the limitations of the measurement tool. Additionally, validated measures were often better designed to reduce social desirability bias in participants’ responses to SI-related questions. For instance, validated tools may have assessed SI more discreetly, thereby reducing the likelihood of participants concealing their true thoughts, which could have contributed to the observed weaker correlation when these tools were used (109).

### 4.5 The moderating role of PIU measurement tools

Similarly, the type of measurement tool used to assess PIU was found to significantly moderate the relationship between PIU and SI. When tools designed to measure mobile phone addiction were employed, the correlation between PIU and SI was comparatively weaker, suggesting that the risk of SI among Chinese adolescents addicted to mobile phones may have been lower than that associated with other forms of internet addiction. Several plausible explanations can account for this observation: To begin with, the nature of mobile phone addiction, which typically involves excessive engagement in social media, instant messaging, and recreational activities, might have provided adolescents with social support and emotional regulation, potentially mitigating the onset of SI (110). In contrast, other forms of internet addiction, such as online gambling or pornography, were likely to result in greater psychological distress and isolation, thereby heightening the risk of SI. Moreover, cultural factors were considered to play a significant role; within the Chinese context, mobile phone use for social interaction and entertainment was widely accepted as a normal aspect of adolescent life. This acceptance might have reduced the negative psychological consequences of excessive use, thereby lowering the risk of SI (111). Lastly, mobile phone addiction may have served as a functional coping strategy for adolescents to escape real-life problems, such as academic pressure or family conflicts. While not a healthy coping mechanism, it might have temporarily diminished the likelihood of developing SI (112–114). Additionally, the specificity of mobile phone addiction measurement tools, which tended to focus on the frequency and duration of use rather than on deeper psychological distress, could have led to an underestimation of the correlation between PIU and SI (115). These considerations suggest that mobile phone addiction among Chinese adolescents may have been associated with a lower risk of SI compared to other types of internet addiction.

### 4.6 The moderating role of sex

Nevertheless, the study results indicated that the relationship between PIU and SI was not significantly moderated by gender. This finding suggested that the impact of PIU on SI remained generally consistent across both males and females. This phenomenon could be attributed to two primary factors: On the one hand, it is plausible that PIU exerted broad and consistent negative effects on mental health that transcended gender differences. Irrespective of gender, excessive or unhealthy internet use could have led to social isolation, sleep disturbances, and emotional instability, all of which might have increased the risk of SI (103, 116, 117). On the other hand, it should be acknowledged that the subgroup analysis in this study included only three articles, which might have limited the generalizability of the findings and the ability to detect a gender moderation effect.

### 4.7 Advantages and limitations

By systematically reviewing studies, this analysis investigated the PIU-suicidal behavior connection in Chinese adolescents. The use of large, varied samples strengthened the conclusions’ reliability. The findings may inform new prevention and management strategies, establishing a solid basis for future interventions.

Nevertheless, certain limitations should be recognized. First, the inclusion of only cross-sectional observational studies meant that the results reflected a correlation between PIU and suicidal behavior, without establishing causality. At the same time, the ongoing debate in the literature regarding the directionality (or bidirectionality) of the relationship between PIU and suicidal behavior was acknowledged, and the incorporation of longitudinal designs in future studies was identified as essential for better understanding these temporal dynamics and causal pathways. Secondly, the significant effects were observed in only a limited number of moderating variables, leaving much of the heterogeneity unaddressed. This limitation indicated that other potential moderating factors influencing the relationship between PIU and suicidal behavior were likely overlooked in the current analysis. Consequently, the full complexity of this relationship may not have been fully captured. It was recommended that future meta-analyses expand their scope to include a wider range of moderating variables, which could lead to a more comprehensive understanding of the factors contributing to variability in outcomes and clarify under which conditions PIU is most strongly associated with suicidal behavior. Thirdly, our analysis was constrained to studies published in Chinese and English, thereby excluding unpublished works, which may have introduced publication bias. Furthermore, by focusing exclusively on Chinese adolescents, the research scope is inherently limited, which restricts the applicability of the findings to broader populations. The exclusion of other age groups, such as children, adults, or elderly individuals, means that the results may not be generalizable beyond the specific demographic studied. Consequently, the insights gained from this research may not fully capture the complexities of PIU and suicidal behavior in different age groups, potentially overlooking important age-related differences that could influence the relationship between these variables. In addition, the reliance on self-report questionnaires across all studies, as opposed to clinical assessments, could have influenced the robustness of the findings. Lastly, a limitation of this study is that subgroup analysis was conducted solely on the relationship between adolescent PIU and SI. This leaves the connections between PIU and SP, as well as SA, underexplored. Future research should conduct subgroup analyses on the relationships between adolescent PIU and SP, as well as SA, to provide a more comprehensive understanding of PIU’s impact on different types of suicidal behavior. Such refined analyses would enable researchers to more accurately identify the underlying mechanisms of various suicidal behaviors, thereby offering stronger evidence for prevention and intervention strategies.

## 5 Conclusion

This study systematically reviewed the relationship between PIU and suicidal behavior among Chinese adolescents. A significant positive correlation between PIU and suicidal behavior was revealed, suggesting that PIU might have been a critical risk factor contributing to suicidal behavior in this population. The findings underscored the substantial impact of internet use on adolescent mental health, with a particular emphasis on the increased risk of SI among adolescents in China’s central and western regions, university students, and those suffering from depressive disorders. These groups were found to face more complex psychological and social pressures, which may have intensified the association between PIU and suicidal behavior. By focusing on these vulnerable groups and their distinct challenges, this study offers valuable insights into tailoring preventive strategies to different contexts and regions, thereby enhancing the effectiveness of interventions. Therefore, it was deemed imperative to implement targeted preventive interventions for these high-risk groups.

Based on these findings, it was recommended that mental health education and interventions for adolescents be strengthened. This should have included the widespread dissemination of mental health knowledge at the secondary and tertiary education levels, focusing on enhancing adolescents’ ability to manage stress and regulate emotions, as well as the establishment of early identification and intervention mechanisms to provide timely psychological support. Secondly, it was suggested that regulations and management of internet use be improved to promote healthy internet habits, particularly among adolescents in the central and western regions and university students, to mitigate the negative psychological impacts of problematic internet use. For high-risk groups, such as those with depressive disorders, specific psychological support and interventions, including behavioral correction, psychotherapy, and family support, were recommended. The relevance of these findings lies in their potential to guide policy-makers and practitioners in designing targeted interventions for populations at higher risk of PIU and suicide, contributing to long-term improvements in adolescent mental health. At the same time, it was recommended that the government introduce relevant policies to support the promotion of mental health education and internet use regulations, especially in resource-constrained areas like central and western China. Furthermore, it was suggested that cooperation among families, schools, and society be enhanced to support healthy internet use and mental health among adolescents. Finally, the establishment of a long-term monitoring mechanism was recommended to continuously track the relationship between PIU and adolescent mental health, providing scientific evidence for policy-making and intervention strategies. The relevance of this study lies in its contribution to addressing a critical public health issue by identifying high-risk groups and proposing specific, evidence-based intervention strategies that can inform mental health policy and practice in China.

## Data Availability

The original contributions presented in the study are included in the article/**Supplementary Material**. Further inquiries can be directed to the corresponding author.
