# Supplementary material for: Correlations between problematic internet use and suicidal behavior among Chinese adolescents: a systematic review and meta-analysis
Source: Front Psychiatry. 2024 Nov 11;15:1484809. doi: 10.3389/fpsyt.2024.1484809 (PMC11586362; doi:10.3389/fpsyt.2024.1484809)
Supplement: Supplementary file 1 [file DataSheet1.docx]

**Additional file 1**

**Supplementary material 1:** Search strategy used in the current systematic review and meta-analysis

**Supplementary material 2:** Methodological quality assessment of the literature

**Supplementary material 3:** Funnel plot of the relationship between problematic internet use and suicide attempts and analysis of publication bias and sensitivity as assessed by the Egger test

**Supplementary material 4:** Funnel plot of the relationship between problematic internet use and suicide plans and analysis of publication bias and sensitivity as assessed by the Egger test

# 1 Supplementary material 1: Search strategy used in the current systematic review and meta-analysis

***PUBMED***

#1

("Suicide"[MeSH] OR suicide[Title/Abstract] OR "suicidal ideation"[MeSH] OR "suicidal ideation"[Title/Abstract]) OR (suicidal behav*[Title/Abstract]) OR (self?harm*[Title/Abstract]) OR (self?injur*[Title/Abstract]) OR (self?poison*[Title/Abstract]) OR (self?inflict*[Title/Abstract])

#2

("Adolescent"[MeSH] OR adolescent[Title/Abstract] OR adolescence[Title/Abstract] OR "teenager"[Title/Abstract] OR "teenagers"[Title/Abstract] OR "youth"[Title/Abstract] OR "young people"[Title/Abstract] OR "high school students"[Title/Abstract] OR "middle school students"[Title/Abstract] OR "college students"[Title/Abstract] OR "university students"[Title/Abstract] OR "students"[Title/Abstract])

#3

(Internet addiction disorder [Mesh])

#4

(cell phone[Title/Abstract]) OR (cell phones[Title/Abstract]) OR (cellular phone[Title/Abstract]) OR (cellular phones[Title/Abstract]) OR (cellular telephone[Title/Abstract]) OR (cellular telephones[Title/Abstract]) OR (mobile devices[Title/Abstract]) OR (mobile phone[Title/Abstract]) OR (smart phone[Title/Abstract]) OR (smartphone[Title/Abstract])

#5

(addiction[Title/Abstract]) OR (dependence[Title/Abstract]) OR (dependency[Title/Abstract]) OR (abuse[Title/Abstract]) OR (addicted to[Title/Abstract]) OR (overuse[Title/Abstract]) OR (problem use[Title/Abstract]) OR (compensatory use[Title/Abstract])

#6

(internet addiction [Title/Abstract]) OR (problematic internet use [Title/Abstract]) OR (internet addiction disorder [Title/Abstract]) OR (pathological internet use [Title/Abstract]) OR (excessive internet use [Title/Abstract]) OR (compulsive internet use [Title/Abstract]) OR (internet dependency [Title/Abstract]) OR (computer addiction [Title/Abstract]) OR (internet use disorder [Title/Abstract])

#1 AND #2 AND (#3 OR (#4 AND #5) OR #6)

**N=176**

***Web of Science***

#1

TS=("Suicide" OR "suicidal ideation" OR "suicidal behav*" OR "self harm*" OR "self injur*" OR "self poison*" OR "self inflict*")

#2

TS=("Adolescent" OR adolescent* OR adolescence OR "teenager" OR "teenagers" OR "youth" OR "young people" OR "high school students" OR "middle school students" OR "college students" OR "university students" OR "students")

#3

TS=("internet addiction disorder" OR (("cell phone" OR "cell phones" OR "cellular phone" OR "cellular phones" OR "cellular telephone" OR "cellular telephones" OR "mobile devices" OR "mobile phone" OR "smart phone" OR "smartphone") AND ("addiction" OR "dependence" OR "dependency" OR "abuse" OR "addicted to" OR "overuse" OR "problem use" OR "compensatory use")) OR ("internet addiction" OR "problematic internet use" OR "internet addiction disorder" OR "pathological internet use" OR "excessive internet use" OR "compulsive internet use" OR "internet dependency" OR "computer addiction" OR "internet use disorder"))

#5

#1 AND #2 AND #3

**N=470**

***EMBASE***

#1

'suicide'/exp OR 'suicide':ti,ab OR 'suicidal ideation'/exp OR 'suicidal ideation':ti,ab OR 'suicidal behav*':ti,ab OR 'self harm*':ti,ab OR 'self injur*':ti,ab OR 'self poison*':ti,ab OR 'self inflict*':ti,ab

#2

'adolescent'/exp OR adolescent*:ti,ab OR adolescence:ti,ab OR 'teenager':ti,ab OR 'teenagers':ti,ab OR 'youth':ti,ab OR 'young people':ti,ab OR 'high school students':ti,ab OR 'middle school students':ti,ab OR 'college students':ti,ab OR 'university students':ti,ab OR 'students':ti,ab

#3

'internet addiction disorder'/exp OR (('cell phone':ti,ab OR 'cell phones':ti,ab OR 'cellular phone':ti,ab OR 'cellular phones':ti,ab OR 'cellular telephone':ti,ab OR 'cellular telephones':ti,ab OR 'mobile devices':ti,ab OR 'mobile phone':ti,ab OR 'smart phone':ti,ab OR 'smartphone':ti,ab) AND ('addiction':ti,ab OR 'dependence':ti,ab OR 'dependency':ti,ab OR 'abuse':ti,ab OR 'addicted to':ti,ab OR 'overuse':ti,ab OR 'problem use':ti,ab OR 'compensatory use':ti,ab)) OR ('internet addiction':ti,ab OR 'problematic internet use':ti,ab OR 'internet addiction disorder':ti,ab OR 'pathological internet use':ti,ab OR 'excessive internet use':ti,ab OR 'compulsive internet use':ti,ab OR 'internet dependency':ti,ab OR 'computer addiction':ti,ab OR 'internet use disorder':ti,ab)

#4

#1 AND #2 AND #3

**N=269**

# 2 Supplementary material 2: Methodological quality assessment of the literature

S2a: Agency for Health Care Research and Quality (AHRQ)

| Study | Year | ① | ② | ③ | ④ | ⑤ | ⑥ | ⑦ | ⑧ | ⑨ | ⑩ | ⑪ |
| --- | --- | --- | --- | --- | --- | --- | --- | --- | --- | --- | --- | --- |
| Yang et al. | 2010 | Y | N | Y | Y | U | N | Y | N | Y | N | N |
| Lin et al. | 2014 | Y | Y | Y | Y | Y | N | Y | Y | Y | N | N |
| Zhang et al. | 2014 | Y | Y | Y | Y | U | N | Y | Y | Y | N | N |
| Wang et al. | 2014 | Y | Y | Y | Y | Y | N | Y | Y | Y | N | N |
| Zhang et al. | 2018 | Y | Y | Y | Y | N | N | Y | Y | Y | N | N |
| Wang et al. | 2019 | Y | Y | Y | Y | Y | Y | Y | Y | Y | Y | Y |
| Lu et al. | 2020 | Y | Y | Y | Y | Y | N | Y | Y | Y | Y | N |
| Kuang et al. | 2020 | Y | Y | Y | Y | Y | Y | Y | Y | Y | Y | N |
| Guo et al. | 2020 | Y | Y | Y | Y | Y | Y | Y | Y | Y | Y | N |
| Yu et al. | 2020 | Y | Y | Y | Y | Y | Y | Y | Y | Y | Y | N |
| Huang et al. | 2020 | Y | Y | Y | Y | Y | Y | Y | Y | Y | Y | N |
| Shen et al. | 2020 | Y | Y | Y | Y | Y | Y | Y | Y | Y | Y | N |
| Pan et al. | 2020 | Y | Y | Y | Y | N | N | Y | Y | Y | N | N |
| Shen et al. | 2020 | Y | Y | Y | Y | Y | Y | Y | Y | Y | Y | N |
| Wen et al. | 2021 | Y | Y | Y | Y | U | N | Y | Y | Y | N | N |
| Chang et al. | 2021 | Y | Y | Y | Y | Y | Y | Y | Y | Y | Y | N |
| Wang et al. | 2022 | Y | Y | Y | Y | N | N | Y | Y | Y | N | N |
| Huang et al. | 2022 | Y | Y | Y | Y | Y | Y | Y | Y | Y | Y | N |
| Junus et al. | 2023 | Y | Y | Y | Y | Y | Y | Y | Y | Y | Y | N |
| Wang et al. | 2023 | Y | Y | Y | Y | Y | Y | Y | Y | Y | Y | N |
| Kang et al. | 2023 | Y | Y | Y | Y | Y | Y | Y | Y | Y | Y | N |
| Cheng et al. | 2024 | Y | Y | Y | Y | Y | Y | Y | Y | Y | Y | N |
| zhang et al. | 2024 | Y | Y | Y | Y | Y | Y | Y | Y | Y | Y | N |

Y: Yes; N: No; U: Uclear; ①Define the source of information (survey, record review)；②List inclusion and exclusion criteria for exposed and unexposed subjects(cases and controls) or refer to previous publications; ③Indicate time period used for identifying patients; ④indicate whether or not subjects were consecutive if not population-based; ⑤indicate if evaluators of subjective components of study were masked to other aspects of the status of the participants; ⑥Describe any assessments undertaken for quality assurance purposes(e.g., test/retest of primary outcome measurements); ⑦Explain any patient exclusions from analysis; ⑧Describe how confounding was assessed and/or controlled; ⑨If applicable, explain how missing data were handled in the analysis; ⑩Summarize patient response rates and completeness of data collection; ⑪Clarify what follow-up, was expected and percentage of patients for which incomplete data or follow-up was obtained.

# 3 Supplementary material 3: Funnel plot of the relationship between problematic internet use and suicide attempts and analysis of publication bias and sensitivity as assessed by the Egger test

S3a: A funnel plot of the relationship between problematic internet use and suicide attempts


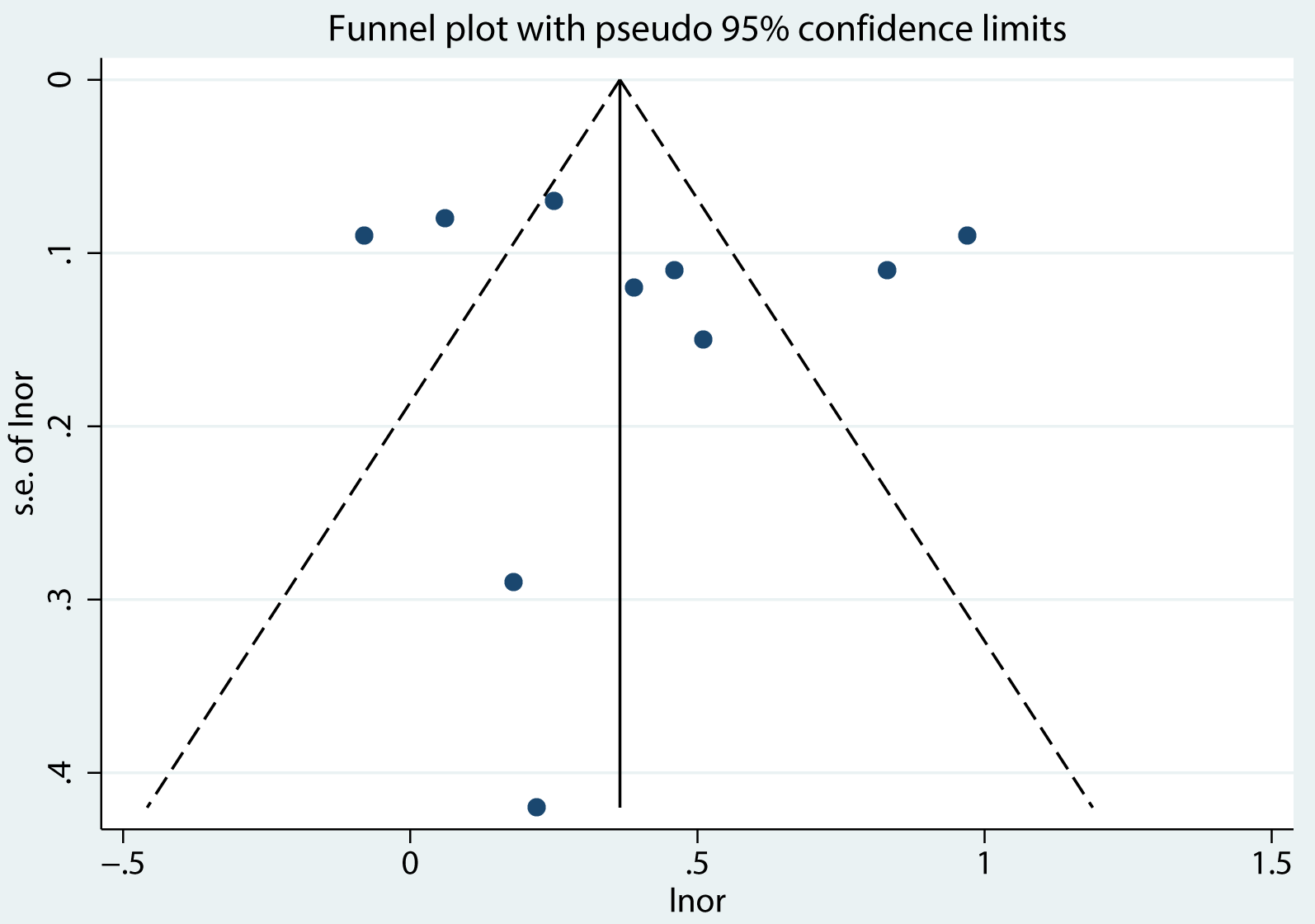


S3b: Publication bias in the Egger test assessment of the relationship between problematic internet use and suicide attempts


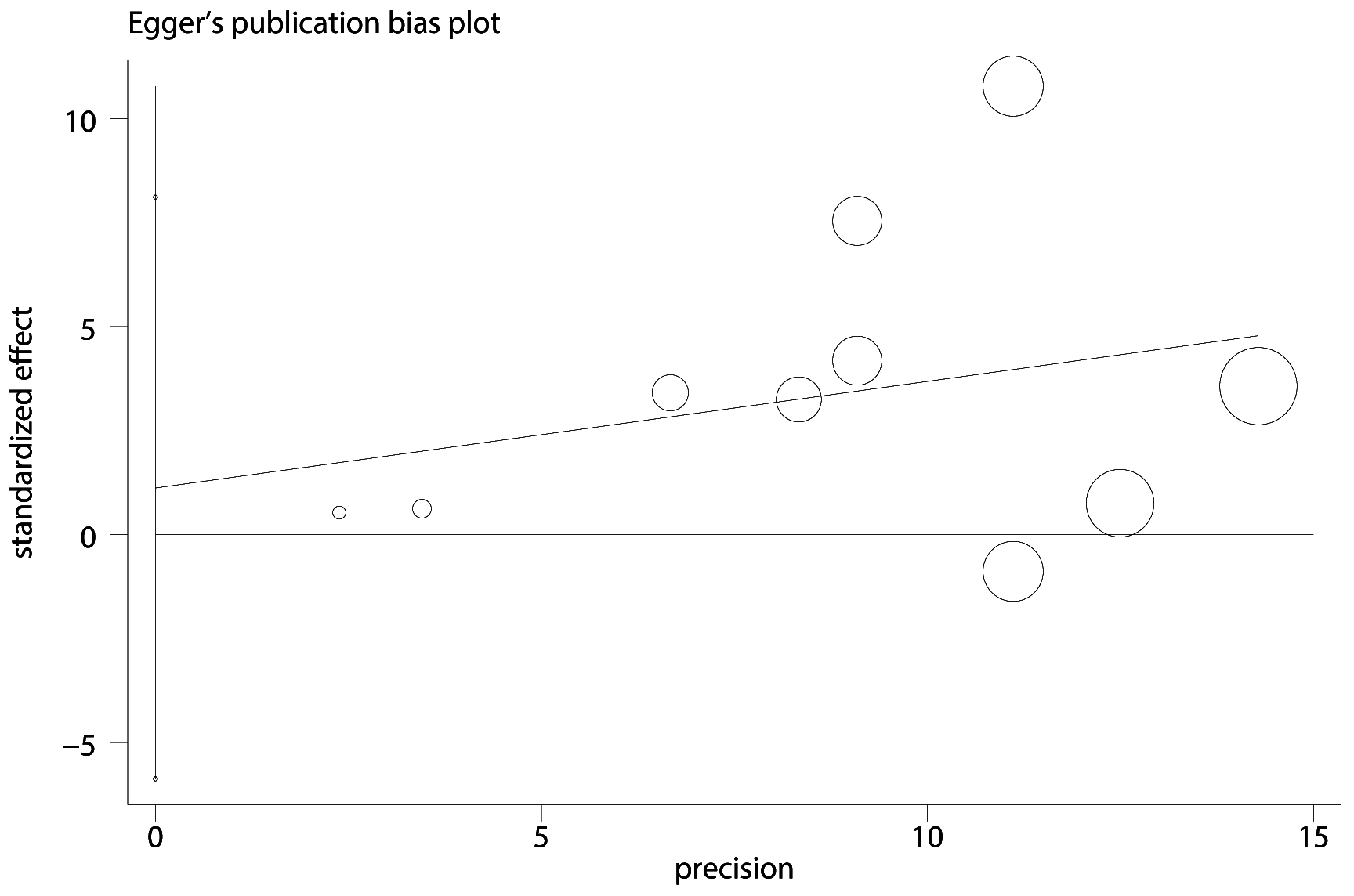


S3c: Sensitivity analysis of the relationship between problematic internet use and suicide attempts


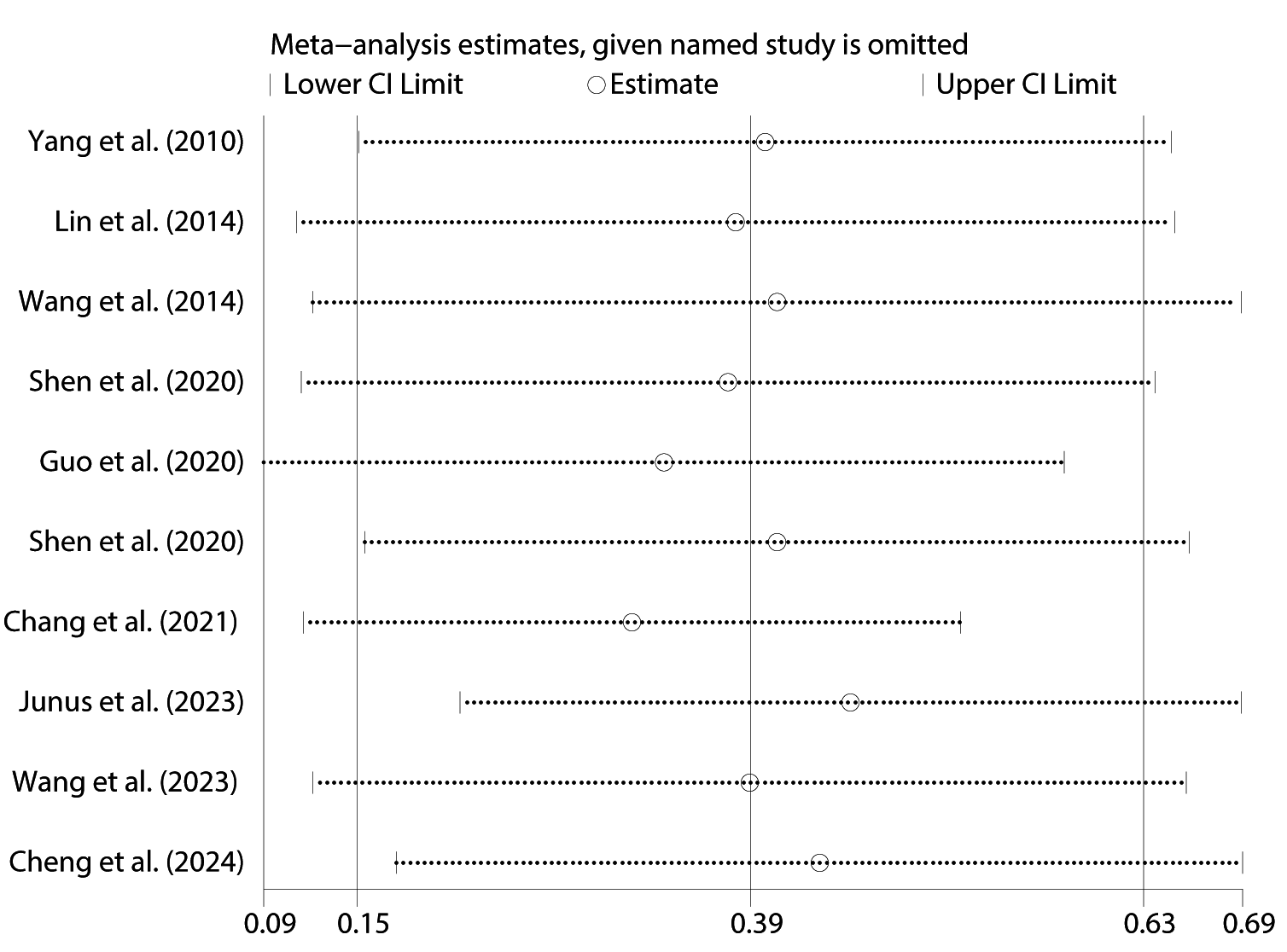


# 4 Supplementary material 4: Funnel plot of the relationship between problematic internet use and suicide plans and analysis of publication bias and sensitivity as assessed by the Egger test

S4a: A funnel plot of the relationship between problematic internet use and suicidal plans


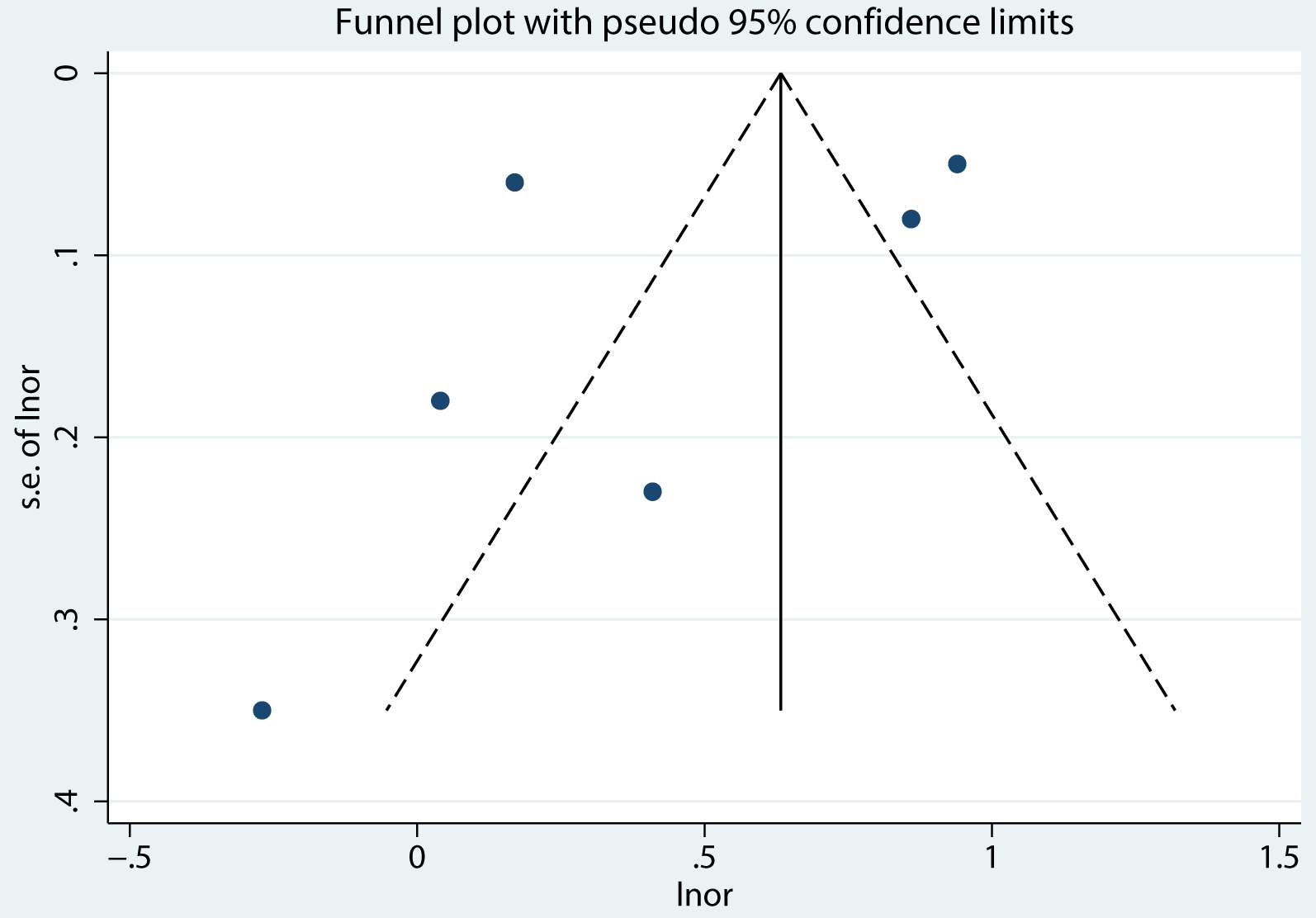


S4b: Publication bias in the Egger test assessment of the relationship between problematic internet use and suicidal plans


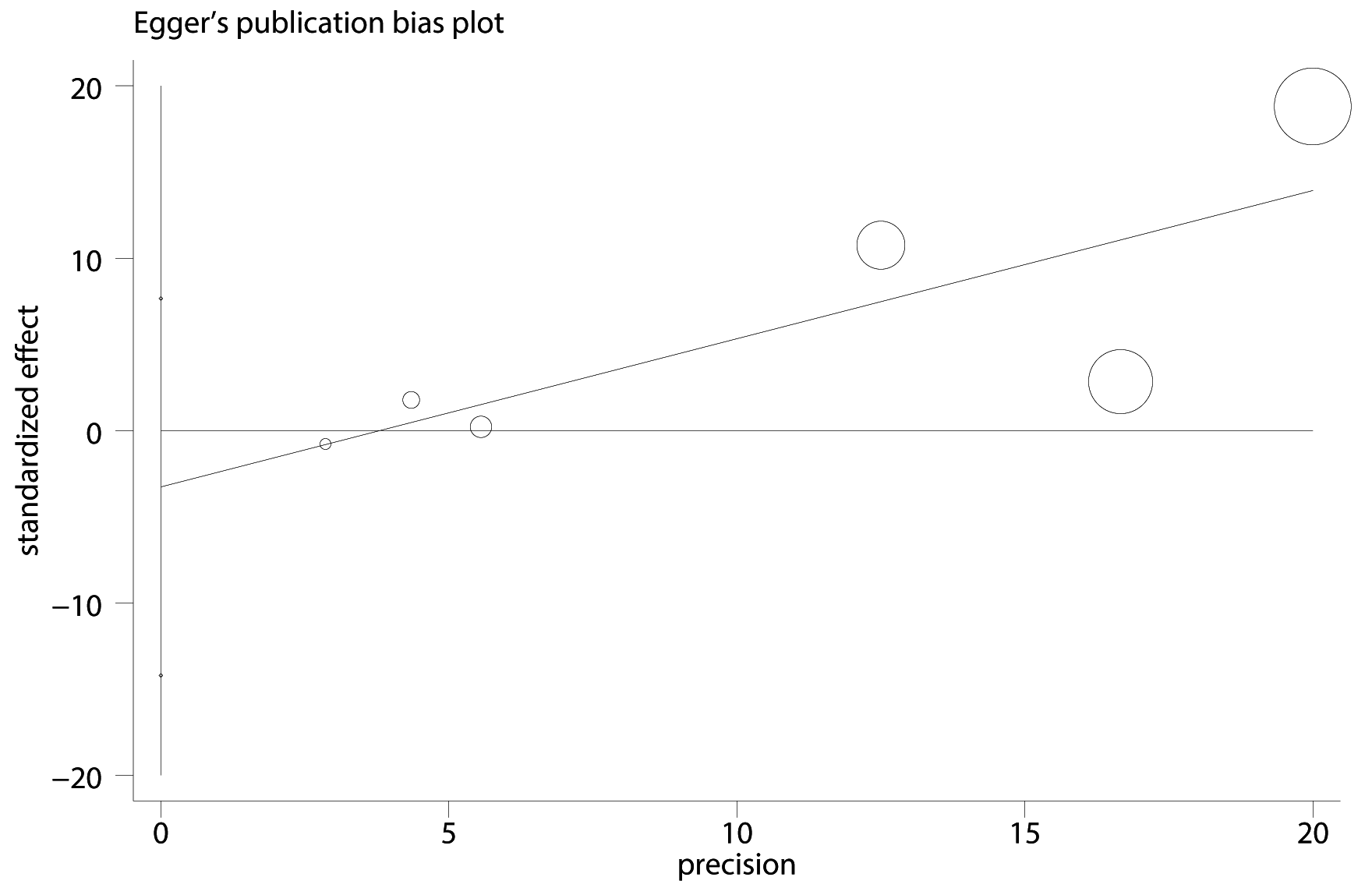


S4c: Sensitivity analysis of the relationship between problematic internet use and suicidal plans

**
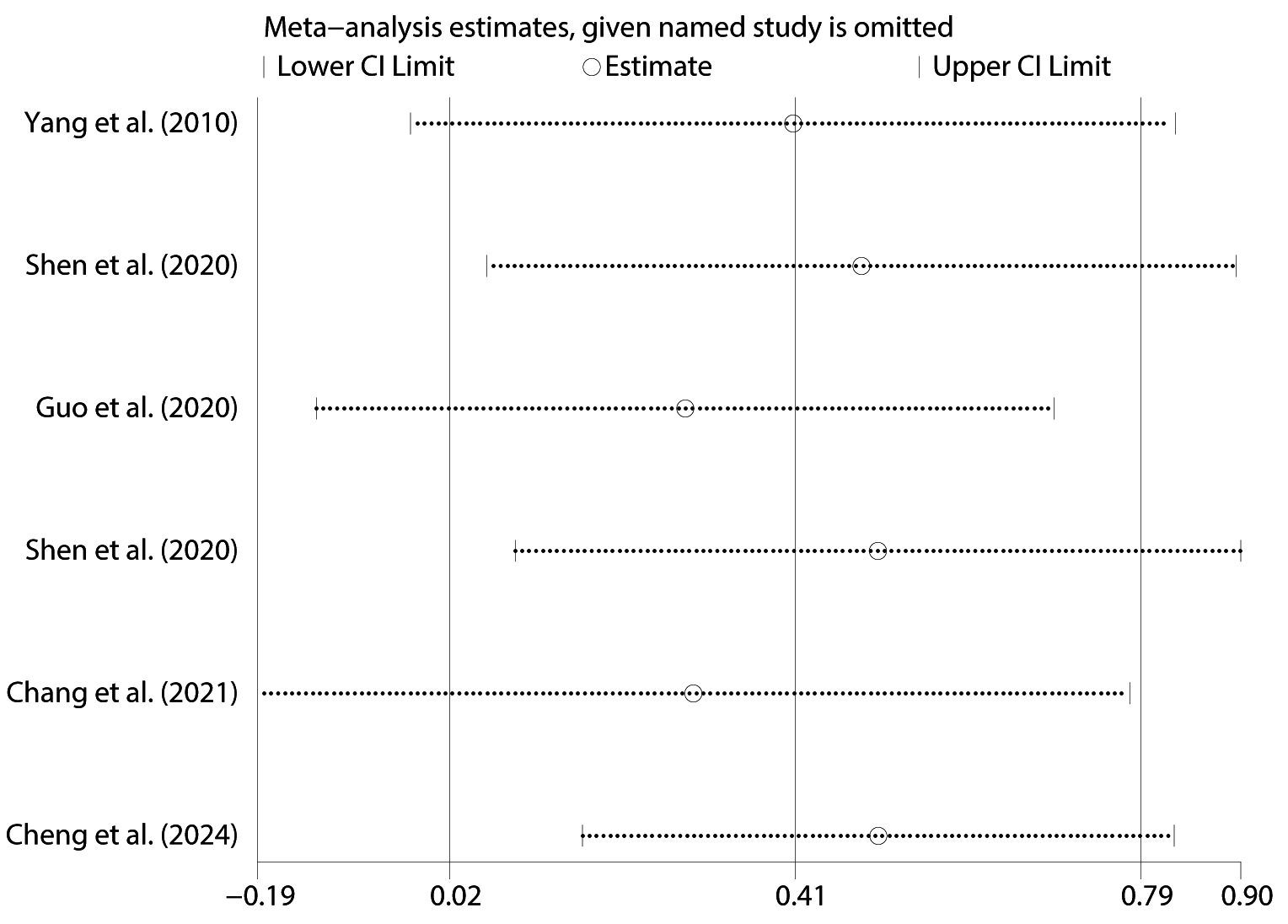
**
